# Imaging the mammary gland and mammary tumours in 3D: optical tissue clearing and immunofluorescence methods

**DOI:** 10.1186/s13058-016-0754-9

**Published:** 2016-12-13

**Authors:** Bethan Lloyd-Lewis, Felicity M. Davis, Olivia B. Harris, Jessica R. Hitchcock, Filipe C. Lourenco, Mathias Pasche, Christine J. Watson

**Affiliations:** 1 Department of Pathology, University of Cambridge, Cambridge, CB2 1QP UK; 2 School of Pharmacy, The University of Queensland, Brisbane, 4072 Australia; 3 Wellcome Trust-Medical Research Council Cambridge Stem Cell Institute, University of Cambridge, Cambridge, CB2 1QR UK; 4 Cancer Research UK Cambridge Institute, University of Cambridge, Li Ka Shing Centre, Cambridge, CB2 0RE UK; 5 Medical Research Council Laboratory for Molecular Biology, Cambridge, CB2 0QH UK

**Keywords:** Mammary gland, Lactation, Breast cancer, Tissue clearing, 3D imaging, Fluorescence microscopy, Light sheet fluorescence microscopy, Two-photon microscopy

## Abstract

**Background:**

High-resolution 3D imaging of intact tissue facilitates cellular and subcellular analyses of complex structures within their native environment. However, difficulties associated with immunolabelling and imaging fluorescent proteins deep within whole organs have restricted their applications to thin sections or processed tissue preparations, precluding comprehensive and rapid 3D visualisation. Several tissue clearing methods have been established to circumvent issues associated with depth of imaging in opaque specimens. The application of these techniques to study the elaborate architecture of the mouse mammary gland has yet to be investigated.

**Methods:**

Multiple tissue clearing methods were applied to intact virgin and lactating mammary glands, namely 3D imaging of solvent-cleared organs, see deep brain (seeDB), clear unobstructed brain imaging cocktails (CUBIC) and passive clarity technique. Using confocal, two-photon and light sheet microscopy, their compatibility with whole-mount immunofluorescent labelling and 3D imaging of mammary tissue was examined. In addition, their suitability for the analysis of mouse mammary tumours was also assessed.

**Results:**

Varying degrees of optical transparency, tissue preservation and fluorescent signal conservation were observed between the different clearing methods. SeeDB and CUBIC protocols were considered superior for volumetric fluorescence imaging and whole-mount histochemical staining, respectively. Techniques were compatible with 3D imaging on a variety of platforms, enabling visualisation of mammary ductal and lobulo-alveolar structures at vastly improved depths in cleared tissue.

**Conclusions:**

The utility of whole-organ tissue clearing protocols was assessed in the mouse mammary gland. Most methods utilised affordable and widely available reagents, and were compatible with standard confocal microscopy. These techniques enable high-resolution, 3D imaging and phenotyping of mammary cells and tumours *in situ*, and will significantly enhance our understanding of both normal and pathological mammary gland development.

**Electronic supplementary material:**

The online version of this article (doi:10.1186/s13058-016-0754-9) contains supplementary material, which is available to authorized users.

## Background

The mammary gland is composed of a branching epithelial ductal network deeply embedded within a vascularised stromal matrix made up of adipocytes, fibroblasts and immune cells [1]. Due to its capacity for rapid growth and regeneration, the mouse mammary gland is a powerful model in which to study a range of developmental processes associated with tissue morphogenesis and remodelling, and provides important insights into the perturbations that give rise to breast cancer [1]. However, visualisation of the complex cellular networks within the intact mammary epithelium is greatly impeded by the lipid-rich and optically opaque nature of this organ. As a result, immunolabelling and fluorescence imaging of mammary tissue has traditionally been performed using thin tissue sections with assumptions about the architectural context and uniformity of a particular 2D plane. Whilst 3D imaging has recently been used to investigate mammary stem cell dynamics [2–4] and binucleated cells in lactational alveoli [5], these studies have relied on tissue microdissection [2, 5] or enzymatic digestion [3]. Consequently, visualisation of the mammary epithelial tree at single-cell resolution within its native stroma remains a fundamental challenge in mammary gland research.

The utility of rendering tissue optically transparent has been appreciated for over a century [6]. However, recent advances in fluorescence microscopy have heralded the development of numerous whole-organ tissue clearing methods aimed at improving optical access and depth of imaging in intact specimens (reviewed in [7]). These methods are primarily based on mitigating light scattering caused by heterogeneous cellular components with different refractive indices (RIs). Techniques broadly rely either on organic solvent-based or hydrophilic reagent-based clearing solutions to homogenise RIs within tissue, and may also include prior hydrogel embedding to stabilise cellular structures. Whilst many protocols were originally optimised for the central nervous system and whole embryos, recent refinements in tissue clearing techniques have facilitated exceptional optical access to many other mammalian tissues. However, the application of these techniques to the mammary gland is yet to be explored.

Here, we describe the application of four leading tissue clearing protocols, namely three-dimensional imaging of solvent-cleared organs (3DISCO) [8], see deep brain (SeeDB) [9], clear unobstructed brain imaging cocktails (CUBIC) [10] and passive clarity technique (PACT) [11], to virgin and lactating mammary glands. Whilst the underlying principles for achieving tissue transparency are fundamentally different in each of these methods, the majority utilise simple and affordable reagents, and can be completed within two weeks.

3DISCO [8] is based on earlier clearing methods that use high-index organic solvents such as benzyl alcohol benzyl benzoate (BABB) [12, 13], and can be combined with optimised whole-mount immunolabelling procedures (termed iDISCO) [14]. To date, 3DISCO remains the only method previously applied to mammary tissue, albeit superficially [8]. PACT [11] relies on hydrogel embedding to stabilise cellular structures prior to tissue delipidation using ionic detergents. Due to the need for custom electrophoresis equipment, CLARITY [15] is difficult to implement and can lead to heat-induced tissue damage and epitope loss [11]. The PACT protocol circumvents these issues, relying instead on passive diffusion. Furthermore, PACT utilises more economical RI matching solutions (RIMS and sRIMS), an additional benefit over CLARITY. CUBIC [10] is a urea-based clearing reagent that includes aminoalcohols and detergents to remove lipids and homogenise RIs within tissue. In addition, CUBIC reagents decolourise blood by eluting the heme chromophore, further enhancing optical transparency by minimising light absorption [16]. Finally, SeeDB [9] is a water-based optical clearing agent that utilises saturated solutions of fructose and alpha-thioglycerol (which limits autofluorescence) for RI matching.

We compared these four protocols for optical transparency and tissue preservation in the intact mammary gland, in addition to their compatibility with immunofluorescent labelling and 3D imaging of mammary epithelial cells. Their suitability for the analysis of mammary tumours was also investigated. Using standard confocal and advanced imaging techniques, ductal and lobulo-alveolar structures could be readily visualised in cleared tissue, with varying degrees of fluorescent signal preservation between the different methods. Overall, our results placed SeeDB and CUBIC as methods of choice for high-resolution fluorescence imaging and whole-mount histochemical staining of mammary glands. The ability to visualise the mammary epithelial tree at single cell resolution within its native stroma will provide invaluable insight into mammary gland development and tumourigenesis.

## Methods

### Reagents and antibodies

The following reagents were purchased from Sigma Aldrich: neutral buffered formalin (NBF), dimethyl sulfoxide (DMSO), tetrahydrofuran (THF), dichloromethane (DCM), dibenzyl ether (DBE), benzyl alcohol, benzyl benzoate, urea, N,N,N’,N’-tetrakis(2-hydroxypropyl)ethylenediamine, 2,2′,2″-nitrilotriethanol, fructose, α-thioglycerol, D-sorbitol and 4′,6-diamidino-2-phenylindole (DAPI) dilactate. RapiClear-CLARITY Specific (RC-CS) Solution and Mounting Medium and iSpacer imaging chambers were purchased from the SunJin Lab: 2,2′-Azobis[2-(2-imidazolin-2-yl)propane] dihydrochloride was purchased from Wako Pure Chemical Industries. Imaging dishes were purchased from Ibidi (81158). Acrylamide (40%) was purchased from Bio-Rad Laboratories. Sodium dodecyl sulphate (SDS) was purchased from Melford Laboratories. Triton-X100 was purchased from VWR International. The following primary antibodies were used for immunostaining: rabbit anti-α-smooth muscle actin (SMA) (Abcam, ab5694; 1:200-1:300 for 2D and 3D studies), rabbit anti-keratin 5 (BioLegend, 905501; 1:100 (3D)), rat anti-cytokeratin 8 (Developmental Studies Hybridoma Bank, TROMA-I; 1:50 (3D) or 1:150-200 (2D)), rabbit anti-E-cadherin (Cell Signaling, 3195; 1:50 (3D) or 1:200 (2D)), mouse anti-E-cadherin (BD Transduction Laboratories, 610182; 1:300 (2D)), rabbit anti-cleaved caspase 3 (Cell Signaling, 9661S; 1:200 (2D)) and rabbit anti-human epidermal growth factor receptor 2 (HER2) (DAKO, A0485; 1:300 (3D) or 1:500 (2D)). The following Alexa Fluor conjugated secondary antibodies were purchased from Life Technologies and diluted 1:500 (2D and 3D studies) in blocking buffer: goat anti-rat Cy3 (A10522), goat anti-rabbit 647 (A21245) and chicken anti-rabbit 647 (A21443). Anti-rabbit horseradish peroxidase (HRP)-conjugated secondary antibody was purchased from DAKO (P0448; 1:500).

### Mice

Mice (C57BL/6 and BALB/c) were housed in individually ventilated cages under a 12:12 h light-dark cycle, with water and food available *ad libitum*. Mice were euthanized by dislocation of the neck or terminal anaesthesia. All tissue from virgin mice was harvested during puberty (5–8 weeks). For studies during lactation, mice were mated with studs, allowed to litter and tissue was harvested between lactation days 2 to 10. Mammary glands (excluding the cervical (first) pair) were excised and immediately spread and fixed on card (Tetra Pak) in 10% NBF for 9 h at room temperature, unless otherwise specified. A 9-h fix provided optimal staining for all antibodies used in this study; however, SMA and K8 also performed well with overnight fixation (4 °C). Fixed tissue was stored at 4 °C in phosphate-buffered saline (PBS) containing sodium azide (0.05% (w/v)) for up to 8 weeks.

Syngeneic mammary tumours were established by orthotopically implanting 5 × 10^3^ TUBO cells [17], into the abdominal (fourth) mammary gland. This cloned cell line was established from a mammary carcinoma that spontaneously arose in a BALB-neuT mouse and therefore carries the Her-2/neu oncogene driven by the MMTV promoter. Mice were monitored regularly and tumours were harvested before exceeding humane endpoints (approx. 4–5 weeks).

### 3DISCO-based clearing and immunohistochemical analysis (IHC)

3DISCO was performed as previously described [8]. Solvent immersion times were adjusted for mammary tissue pieces (approx. 10 × 10 × 1 mm) as follows: 50% (v/v) THF in H_2_O (40 min), 70% (v/v) THF in H_2_O (40 minutes), 80% (v/v) THF in H_2_O (40 minutes), 100% (v/v) THF (3 × 40 minutes), and DCM (15 minutes). All solvent immersion steps were performed in glass vials. Although DBE is reported to be a superior optical clearing agent vs. BABB [8], this solvent requires specialised imaging chambers and solvent-resistant adhesive (e.g. dental cement), and can severely damage objectives if the chamber fails. Thus, we used BABB as the final clearing agent for transmission and confocal imaging in this study. BABB was prepared as a mixture of benzyl alcohol and benzyl benzoate (1:2). Immunostaining was performed as per the iDISCO protocol [14], with a methanol pre-treatment. Following the iDISCO immunolabelling protocol, samples were washed in PBS and incubated with DAPI (10 μM) for 2–3 h, cleared using the 3DISCO protocol and imaged the same day.

### PACT-based clearing and IHC

The A4P0 hydrogel formulation was selected for PACT-based clearing of the mouse mammary gland [11]. A4P0 was prepared to contain acrylamide (4% (v/v)), 2,2′-Azobis[2-(2-imidazolin-2-yl)propane] dihydrochloride (0.25% (w/v)) in PBS. PACT-clearing solution consisted of SDS (8% (w/v)) in distilled water, pH 7.5. Mammary tissue pieces (approx. 10 × 10 × 1 mm) were incubated in A4P0 hydrogel monomer for 4 days at 4 °C and heated to 37 °C in a water bath for 4–6 h. Excess gel was carefully removed from the tissue and samples were immersed in PACT clearing solution for 24 h at room temperature. Samples were immersed in fresh clearing solution, incubated at 37 °C for 4 days (with replenishment every second day), and finally washed with PBS containing triton-X100 (0.1% (w/v)) for 24 h. For immunostaining, samples were blocked in PBS containing triton-X100 (0.5% (w/v)) with goat serum (10% (v/v)) overnight at 4 °C. Primary antibodies were diluted in blocking buffer at 4 °C for 4 days with agitation, tissue was washed (3 × 1 h) in PBS and incubated with Alexa Fluor conjugated secondary antibodies for 2 days. Samples were washed in PBS and incubated with DAPI (10 μM) for 2–3 h. PACT-sRIMS samples were incubated in sRIMS for 4 days or until imaging. sRIMS was prepared by combining sorbitol (70% (w/v)) in 0.02 M phosphate buffer [11, 18]. PACT-RC samples were incubated in Rapiclear CS for 4 h and mounted between two coverslips using RC-CS Mounting Medium and iSpacers for image acquisition and long-term storage.

### CUBIC-based clearing and IHC

CUBIC-based tissue clearing was performed as previously described [10], with minor modifications. CUBIC Reagent 1 was prepared as a mixture of urea (25% (w/w)), N,N,N’,N’-tetrakis(2-hydroxypropyl)ethylenediamine (25% (w/w)), triton-X100 (15% (w/w)) in distilled water. CUBIC Reagent 2 was prepared using sucrose (44% (w/w)), urea (22% (w/w)), 2,2′,2″-nitrilotriethanol (9% (w/w)), triton-X100 (0.1% (w/w)) in distilled water. CUBIC Reagent 1A was prepared using urea (10% (w/w))), N,N,N’,N’-tetrakis(2-hydroxypropyl)ethylenediamine (5% (w/w)), triton-X100 (10% (w/w)) and NaCl (25 mM) in distilled water (unpublished, protocol available at http://cubic.riken.jp/). Tissue pieces (approx. 10 × 10 × 1 mm) were immersed in CUBIC Reagent 1 or 1A at 37 °C for 2–3 days, depending on the size of the tissue piece. For immunostaining samples were washed and subsequently blocked in PBS containing triton-X100 (0.5% (w/v)) and goat serum (10% (v/v)) overnight at 4 °C. Primary antibodies were diluted in blocking buffer at 4 °C for 4 days with gentle rocking. Tissue was washed (3 × 1 h) and incubated with Alexa Fluor conjugated secondary antibodies for 2 days, washed in PBS and incubated with DAPI (10 μM) for 2–3 h. Samples were transferred to CUBIC Reagent 2 at 37 °C for at least 1 day for refractive index matching. Samples were immersed in CUBIC Reagent 2 for imaging and were imaged within 1 week. Diffuse, non-specific fluorescence was observed using the CUBIC protocol in the absence of the primary antibodies (Additional file 1: Figure S1, top panel).

### Whole-mount histochemical and IHC analysis combined with CUBIC-based tissue clearing

Excised and fixed mammary glands were immersed in reagent 1 for 2–3 days. Glands were removed and stained with methyl green (0.5% (w/v)), Harris haematoxylin (10%) or carmine for 1.5–2 h at room temperature with gentle agitation. After staining, tissues were rinsed twice in tap water and once in distilled water before de-staining with acid alcohol (50% ethanol with hydrochloric acid (25 mM)) for 20 minutes and immersion in reagent 2. For detection of β-glucosidase expression (magenta histochemical stain) [19], mammary glands were excised and fixed for 4 h at room temperature. Endogenous β-glucosidase activity was heat inactivated at 65 °C for 15 minutes in PBS. Whole mammary glands were incubated for 48 h at 50 °C in a solution containing 1 part Solution A (5-Bromo-6-chloro-3-indolyl- β-D-glucopyranoside (1% (w/v)) in DMSO) and 25 parts solution B (magnesium chloride (0.02% (w/v)), potassium ferricyanide (0.096% (w/v)) and potassium ferrocyanide (0.13% (w/v)) in PBS). Mammary glands were post-fixed in 10% NBF overnight at 4 °C and cleared using the standard CUBIC-clearing protocol.

For whole-mount immunohistochemical analysis, samples were dehydrated by a methanol series and incubated overnight in methanol containing DMSO (20%) and H_2_O_2_ (3%) to quench endogenous peroxidase activity [20]. Samples were rehydrated by methanol series and blocked and permeabilised in PBS containing BSA (10% (w/v)) and triton-X100 (1% (w/v)). Samples were incubated with rabbit anti-SMA antibody (1:200) for 4 days at 4 °C with gentle agitation. After washing, samples were incubated with anti-rabbit HRP-conjugated secondary antibody (1:500) for a further 2 days, before immersion in reagent 2. Alternatively, quenching, blocking and antibody steps can be performed after immersion in reagent 1 with a similar outcome.

### SeeDB-based clearing and IHC

SeeDB-based clearing was performed as previously described [9], with minor modifications. Briefly, mammary tissue pieces (approx. 10 × 10 × 1 mm) were blocked and permeabilised overnight at 4 °C in PBS with triton-X100 (1% (w/v)) and BSA (10% (w/v)). Primary antibodies were diluted in blocking buffer at 4 °C for 4 days with gentle rocking. Tissue was washed (3 × 1 h) and incubated with secondary antibodies for 2 days before further washing in PBS and incubation with DAPI (10 μM) for 2–3 h. Samples were serially incubated for 8–16 h (twice daily changes) in 2–3 mL of 20%, 40%, 60% and 80% (w/v) fructose in distilled water, and subsequently transferred to 100% (w/v) fructose solution (24 h) and 115% (w/v) fructose solution for 24 h or until imaging. All fructose solutions contained α-thioglycerol (0.5% (v/v)) to inhibit the Maillard reaction [9, 21] and incubations were performed with gentle agitation. For optimal performance, samples were imaged within 2 weeks of clearing; however, staining was still observed up to 6 months after clearing. Diffuse, non-specific fluorescence was observed using the SeeDB protocol in the absence of the primary antibodies (Additional file 1: Figure S1, bottom panel).

### Two-dimensional IHC on CUBIC-recovered and SeeDB-recovered tissue, and formalin-fixed paraffin-embedded (FFPE) tumour sections

Samples were immersed in PBS for approx. 3 days for passive rehydration and removal of the clearing agent [9]. Standard protocols for paraffin processing and embedding using alcohol and xylene were employed. Paraffin-embedded sections (4–6 μm) were de-waxed in xylene and antigen retrieval was performed by boiling in a pressure cooker in tri-sodium citrate buffer (10 mM, pH 6.0), for 11 minutes [22]. Sections were blocked in goat serum (5% (v/v)) in PBS supplemented with triton-X100 (0.05% (w/v)) for 1 h in a humidified chamber at room temperature. Sections were incubated with primary antibodies overnight at 4 °C. Primary antibodies used were: rat anti-K8, rabbit anti-SMA, rabbit anti-E-cadherin, mouse anti-E-cadherin and rabbit anti-cleaved caspase 3. Alexa Fluor conjugated secondary antibodies were diluted 1:500. Nuclei were counterstained with DAPI (1–5 μM).

### Optical clearing and measurement of sample size changes

Mammary tissue pieces were processed using 3DISCO, PACT-RC, PACT-sRIMS, CUBIC or SeeDB-based tissue clearing protocols, and images were acquired on a dissecting microscope (Leica MZ75) with constant exposure, gain and magnification. For quantification of sample size changes, image thresholding was performed using ImageJ (v1.49p, National Institutes of Health) and the pixel area was measured [9]. Volume changes were calculated as the ratio of the pixel area before and after tissue clearing.

### Confocal microscopy

Tissues cleared by 3DISCO, PACT-sRIMS, CUBIC and SeeDB were imaged in their respective RI matching solutions in Ibidi μ-Dishes. PACT-RC-cleared tissues were mounted using iSpacer chambers in RC-CS Mounting Medium. Images were acquired using a Leica TCS SP8 inverted confocal microscope with 10×/0.4 or 20×/0.75 HC PL APO objective lenses. Laser power and gain were adjusted manually to give optimal fluorescence intensity for each fluorophore with minimal photobleaching. Step size and line averaging were kept constant for all main figures (line averaging, 16; step size 1–2), excluding CUBIC and SeeDB depth cueing examples. Imaging depths were recorded from the top of the epithelial structure being imaged (typically 350 μm through the native fat pad for the CUBIC and SeeDB protocols). Image reconstructions were generated using Imaris image management software (v8.0, Bitplane) or ImageJ (v1.50c, National Institutes of Health) [23, 24]. Depth coding was performed using the Temporal Colour Code plugin with the spectrum LUT. De-noising of 3D image sequences was performed in MATLAB (R2014a, The Mathworks Inc.) [25].

### Two-photon and light sheet fluorescence microscopy (LSFM)

Two-photon imaging was performed on a LaVision BioTec TriM Scope II with a 25×/1.05 water dipping lens and an insight DeepSee dual-line laser (tuneable 710–1010 nm and fixed 1040 nm lines), with 810 nm wavelength used to excite DAPI and HER2-AF647. Tiled images, having a 20% overlap, were stitched together using the Grid Collection/Stitching plugin in ImageJ [26].

For LSFM, samples were immunostained and cleared according to the CUBIC protocol [10]. After clearing, samples were embedded within an agarose (1% (w/v) in H_2_O) tube, prepared by aspirating agarose (37–38 °C) into a pre-warmed 1-mL syringe in which the syringe neck had been cut off. Mammary tissue strips were quickly placed within the agarose tube using forceps and centred by rolling the syringe between the palms. After setting, the plunger was removed and the entire syringe was submerged in CUBIC reagent 2. Samples were imaged in reagent 2 or glycerol in H_2_O (34% (w/w)).

The light sheet system was a home-built modified version of the OpenSPIM system [27]. The microscope was built and operated in the T-SPIM layout, whereby illumination happens from two sides simultaneously by overlapping two individual sheets to allow a more even illumination and to reduce artefacts, such as striping. We used two Olympus 5×/0.15 air lenses to generate the light sheet. The higher refractive indices, long working distance (20 mm) and the fact that the lenses were on threaded mounts allowed us to adjust the point of focus accordingly. The imaging light path was equipped with a Nikon 16×/0.8 water dipping lens. We imaged onto an Andor Neo 5.5 (ANDOR) or a Hamamatsu ORCA-Flash4 V2 (Hamamatsu) with 6.5 um pixels. For excitation a home-built laser combiner was used, bundling 405 nm, 488 nm, 561 nm and 640 (Coherent Cube 405 and 640, Coherent Sapphire 488 and 561) into a single-mode fibre. Channels were acquired sequentially and emission was filtered by suitable band-pass or long-pass filters (DAPI: 447/60; AF647: 705/72; both AHF Analysentechnik). The sample was mounted in a 4D (xyzθ) stage (Picard Industries) allowing optimal positioning of the sample in the light sheet. During imaging the sample was moved through the light sheet with a step size of 1.5 μm and the light sheet thickness was adjusted to be ca. 6 μm to warrant an even thickness of the sheet across the entire sample width. Exposure times were between 15 and 150 ms.

## Results

### Optical transparency in the mammary gland

**Fig. 1:**
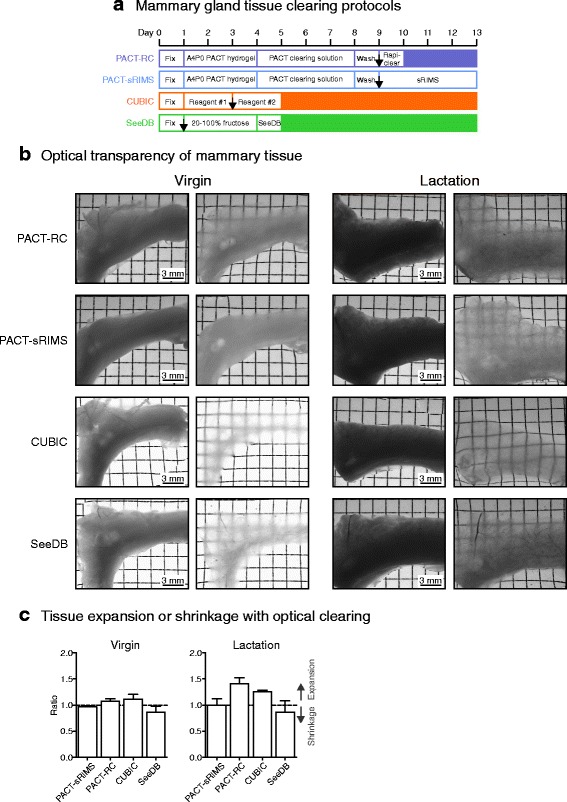
Optical clearing of mammary tissue. **a** Experimental procedure and timelines for optical clearing of mouse mammary tissue. *Black arrows* show the stage at which (optional) immunostaining may be performed. The experimental timeline can be altered depending on the size and nature of the tissue, and the degree of transparency required. **b** Transmission images of whole abdominal (fourth) mammary glands (virgin and lactating) before and after clearing using the passive clarity technique (*PACT*) with rapiclear (*RC*) or sorbitol refractive index matching solution (*sRIMS*), clear unobstructed brain imaging cocktails (*CUBIC*) or the see deep brain (*SeeDB*) clearing protocols. **c** Volume changes resulting from optical clearing of virgin and lactating mammary tissue. Values are representative of measurements from three tissue pieces from each clearing protocol at each developmental time point. See additional file 18 for a high resolution version of these PDFs and http://rdcu.be/lT3Z for additional high resolution examples of these imaging techniques [38]

We evaluated four passive whole-organ tissue clearing protocols for optical clarity and morphology preservation during two distinct phases of mammary gland development, puberty and lactation. The PACT protocol was evaluated using two RI matching solutions: the commercially available aqueous-based solution RapiClear CS® (RI = 1.45) and a more-economical sorbitol-based solution, sRIMS (RI = 1.46) [18]. Of the four methods tested, PACT-based protocols [11] were the most time-intensive and labour-intensive, taking between 10 and 13 days for completion (Fig. 1a). In contrast, the simple, immersion-based tissue clearing protocols CUBIC [10] and SeeDB [9] required only 5 days (Fig. 1a), and resulted in superior optical clarity in both virgin and lactating tissue (Fig. 1b). CUBIC clearing provided the highest degree of transparency in mammary tissue and was also highly effective in decolourising blood vessels (Fig. 1b) [16]. To determine whether these methods altered the structural integrity of mammary tissue, as has been reported for other clearing protocols [12, 28], we measured the sample volume of fixed mammary tissue before and after tissue clearing protocols were applied (Fig. 1c). PACT-RC and CUBIC were associated with a moderate degree of tissue expansion, which was more prominent during lactation. A small reduction in sample volume was observed with SeeDB (Fig. 1c).

We also tested the solvent-based tissue clearing protocol 3DISCO (Additional file 2: Figure S2a), a method previously employed for tissue clearing in the mammary gland [8]. Using 3DISCO, we observed shrinkage of both virgin and lactating mammary tissue samples, which was associated with significant structural deformations to ducts and lactational alveoli (Additional file 2: Figure S2b-d). Although this protocol was extremely rapid to implement (Additional file 2: Figure S2a), achieved a high degree of optical clarity (Additional file 2: Figure S2b) and may be useful in other organ systems [8], its application to the mammary gland, which was left brittle and damaged from the clearing process, is extremely limited. Additionally, the solvents used in this protocol pose significant laboratory safety risks and require specialised imaging chambers [8]. For these reasons we did not pursue 3DISCO further for optical clearing of mammary tissue.

### PACT-based tissue clearing and 3D imaging of the unsectioned mouse mammary gland

**Fig. 2:**
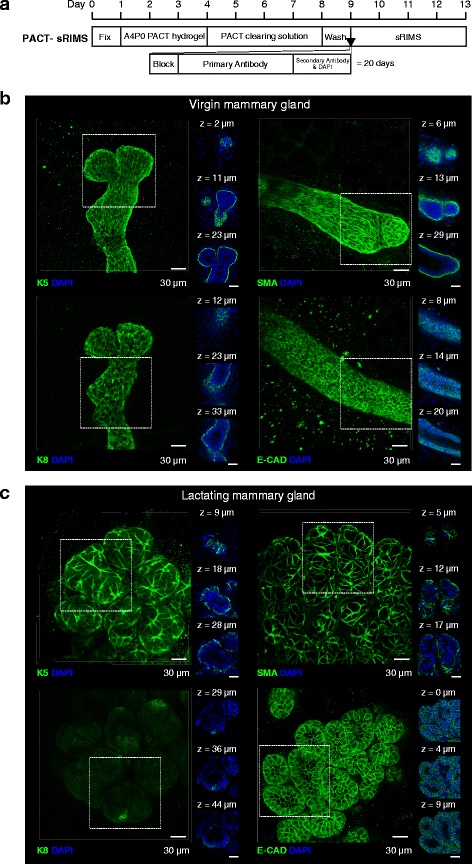
Passive clarity technique (*PACT*)-sorbitol refractive index matching solution (*sRIMS*) clearing and 3D imaging of virgin and lactating mouse mammary tissue. **a** PACT-sRIMS tissue clearing and immunostaining protocol and timeline. Three-dimensional confocal imaging of PACT-sRIMS-cleared virgin (**b**) and lactating (**c**) mammary glands immunostained with basal cell markers (K5 and smooth muscle actin (*SMA*)) and luminal cell markers (K8 and E-cadherin (*E-CAD*)). *Main image* shows the maximum intensity projection of the entire image sequence, with thin optical slices (1 μm) and their depth (*z* value) relative to the first image in the image sequence. These images are representative of images from at least two mice; further examples of PACT-sRIMS-cleared tissue are shown in Additional file 6: Figure S6). *DAPI* 4′,6-diamidino-2-phenylindole. See additional file 18 for a high resolution version of these PDFs and http://rdcu.be/lT3Z for additional high resolution examples of these imaging techniques [38]

We assessed PACT-based clearing approaches for the visualisation of mammary epithelial cells in virgin and lactating mammary glands *in situ*. When combined with whole-mount immunostaining, these protocols required 17 (PACT-RC) to 20 (PACT-sRIMS) days preparation prior to imaging (Additional file 3: Figure S3a and Fig. 2a) [11]. Samples prepared using the PACT-RC protocol are not stable in the RI matching solution, and thus require mounting in a specialised mounting medium (SunJin Labs). Whilst this approach is conducive to long-term sample storage, the limited working distances of standard confocal microscope objectives makes imaging of PACT-RC-mounted samples problematic, as samples cannot be re-orientated against the coverglass for optimal sample illumination (Additional file 3: Figure S3b-c). Consequently, we chose to pursue PACT-sRIMS for whole-mount immunostaining and 3D imaging in this study. However, we note that this is purely a hardware issue, and PACT-RC may be useful with more specialised imaging objectives [18].

PACT-sRIMS permitted imaging of surface structures (Fig. 2b) at marginally improved depths over uncleared tissue (Additional file 4: Figure S4). Using PACT-sRIMS combined with 3D de-noising algorithms (Additional file 5: Figure S5) [25], we were able to visualise surface epithelial structures in 3D at high cellular resolution (Fig. 2b, c and Additional file 6: Figure S6). Figure legends provide a link to higher resolution files. Lactating tissue was particularly amenable to 3D imaging, due to the lower content of adipocytes and the increased surface epithelial mass (Fig. 2c). Using this approach, we were able to observe K5-expressing and SMA-expressing basal cells and K8-expressing and E-cadherin-expressing luminal cells, with confidence.

### CUBIC tissue clearing and 3D imaging of the unsectioned mouse mammary gland

**Fig. 3:**
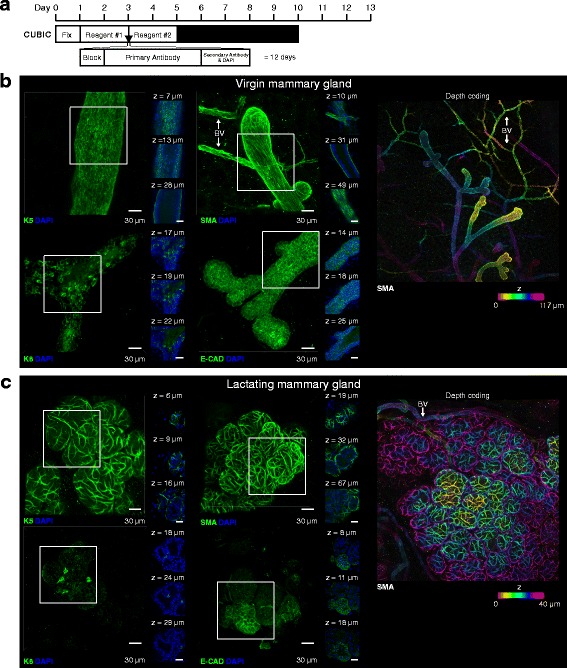
Clear unobstructed brain imaging cocktails (CUBIC) clearing and 3D imaging of virgin and lactating mouse mammary tissue. **a** CUBIC tissue clearing and immunostaining protocol and timeline. Three-dimensional confocal imaging of CUBIC-cleared virgin (**b**) and lactating (**c**) mammary glands immunostained with basal cell markers (K5 and smooth muscle actin (*SMA*)) and luminal cell markers (K8 and E-cadherin (*E-CAD*)). *Main image* (*green*) shows the maximum intensity projection of the entire image sequence, with thin optical slices (1 μm) and their depth (*z* value) relative to the first image in the image sequence. *Right panel* shows depth-coding of SMA-expressing cells; images in an image stack are assigned a colour based on their relative depth. These images are representative of images from more than three mice; further examples of CUBIC-cleared tissue are shown in Additional file 8: Figure S7 and a modified (Reagent 1A) CUBIC protocol in Additional file 9: Figure S8). *BV* blood vessel (SMA-expressing). *DAPI* 4′,6-diamidino-2-phenylindole. See additional file 18 for a high resolution version of these PDFs and http://rdcu.be/lT3Z for additional high resolution examples of these imaging techniques [38]

Like PACT-sRIMS, CUBIC clearing allowed visualisation of the virgin and lactating mammary epithelia at high cellular resolution (Fig. 3, Additional file 7: Movie 1 and Additional file 8: Figure S7). Additionally, due to the high degree of optical transparency achieved by CUBIC clearing, deeper structures could be readily visualised and imaged using this method (Fig. 3). Whilst we observed strong immunostaining with K5 and SMA antibodies using the CUBIC method, K8 and E-cadherin were not readily and uniformly observed in these conditions. This could be due to sub-optimal fixation or a result of differential protein loss caused by exposure to high levels of detergent without prior sub-cellular stabilisation using a hydrogel monomer. However, using a recent modification to the CUBIC Reagent 1 formulation (Reagent 1A, see “Methods”), we observed improved immunostaining of K8 and E-cadherin (Additional file 9: Figure S8), suggesting that epitope availability is better preserved with this new reagent. The stability of various genetically encoded fluorescent proteins (FPs), including GFP, YFP and RFP, was also assessed and found to be adequately preserved through CUBIC processing (Additional file 10: Figure S9a, b).

**Fig. 4:**
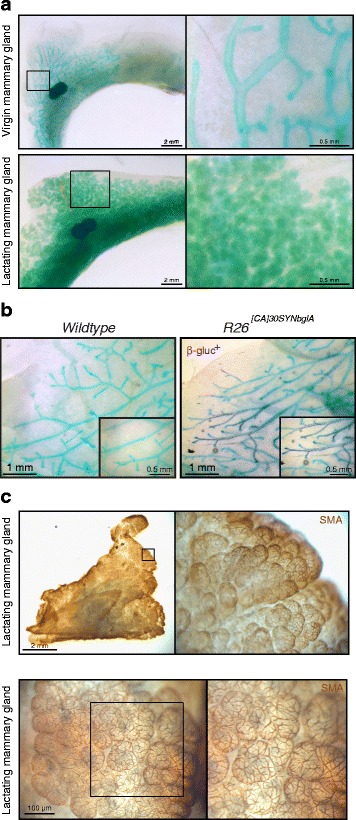
Clear unobstructed brain imaging cocktails (*CUBIC*) clearing for whole-mount transmission imaging of the mouse mammary gland. **a** Virgin and lactating mammary glands stained with methyl green and cleared with CUBIC for whole-mount morphometric analysis. These images are representative of images from more than three mice. **b** Methyl green counterstaining, showing the compatibility of this light green counterstain with magenta-glu detection of β-glucosidase^+^ cells; β-glucosidase^+^ cells are interspersed with unlabelled cells in this *R26* ^*[CA]30SYNbglA*^ mouse model. **c** Compatibility of CUBIC clearing with smooth muscle actin (*SMA*)-immunostaining and horseradish peroxidase-3,3-diaminobenzidine detection. Immunostaining steps can be performed before CUBIC clearing (*top panel*) or after CUBIC clearing (*bottom panel*). See additional file 18 for a high resolution version of these PDFs and http://rdcu.be/lT3Z for additional high resolution examples of these imaging techniques [38]

We determined that CUBIC-cleared samples were amenable to rehydration, paraffin embedding and immunohistochemical staining following whole-mount imaging (Additional file 10: Figure S9c), facilitating the subsequent 2D cellular analysis of whole-mount-imaged mammary structures. On account of the high level of optical clarity achieved using CUBIC-based tissue clearing and its compatibility with subsequent 2D imaging, we also evaluated its utility for whole-mount histochemical analysis (Fig. 4). Currently, carmine is the most prevalent histochemical stain for assessing mammary gland morphogenesis in whole mount. This pigment stains the mammary epithelia an intense pink/red colour and relies on the solvent xylene or methyl salicylate for subsequent optical transparency [29, 30]. Here, we developed and optimised an alternative histochemical stain for the mammary gland, using the cationic dye methyl green and CUBIC tissue clearing. Using this approach we were able to achieve delicate green/blue staining of epithelial structures in the mammary gland of virgin and lactating mice (Fig. 4a). This new staining approach offers improved colour palette flexibility for dual-colour staining, for example with the novel magenta β-glucosidase (SYNbglA) reporter [19] (Fig. 4b). CUBIC clearing was also semi-compatible with the more traditional whole-mount histochemical stains carmine and haematoxylin (Additional file 11: Figure S10), although these were not the focus of this study and staining may be improved with further optimisation. Importantly, CUBIC clearing was compatible with whole-mount DAB immunohistochemical analysis (Fig. 4c), which has not previously been achieved in the mammary gland.

### SeeDB tissue clearing for 3D imaging of the unsectioned mouse mammary gland

**Fig. 5:**
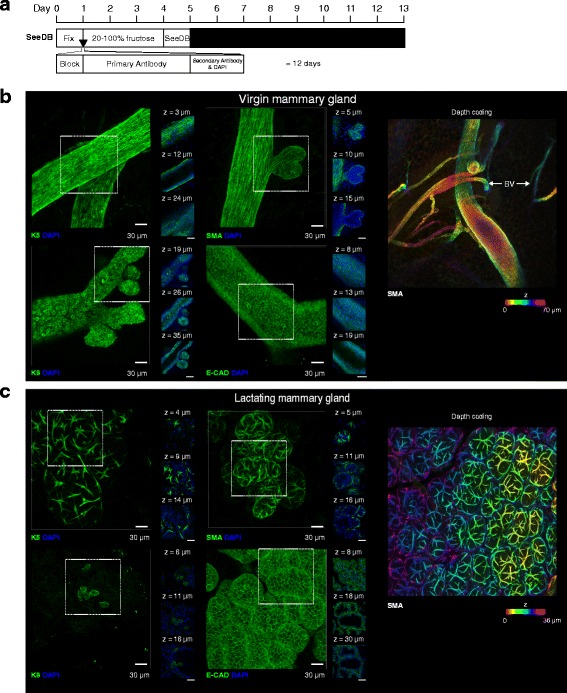
See deep brain (*SeeDB*)-clearing and 3D imaging of virgin and lactating mouse mammary tissue. **a** SeeDB tissue clearing and immunostaining protocol and timeline. Three-dimensional confocal imaging of SeeDB-cleared virgin (**b**) and lactating (**c**) mammary glands immunostained with basal cell markers (K5 and smooth muscle actin (*SMA*)) and luminal cell markers (K8 and E-cadherin (*E-CAD*)). *Main image* (*green*) shows the maximum intensity projection of the entire image sequence, with thin optical slices (1 μm) and their depth (*z* value) relative to the first image in the image sequence. *Right panel* shows depth-coding of SMA-expressing cells; images in an image stack are assigned a colour based on their relative depth. These images are representative of images from more than three mice; further examples of SeeDB-cleared tissue are shown in Additional file 13: Figure S11). *BV* blood vessel (SMA-expressing). *DAPI* 4′,6-diamidino-2-phenylindole. See additional file 18 for a high resolution version of these PDFs and http://rdcu.be/lT3Z for additional high resolution examples of these imaging techniques [38]

Similar to CUBIC clearing, mammary ducts and alveoli could be imaged at high cellular resolution and at considerable depths using SeeDB clearing (Fig. 5, Additional file 12: Movie 2 and Additional file 13: Figure S11). SeeDB-cleared mammary tissue was highly compatible with all antibodies (Fig. 5) and endogenous fluorochromes tested (Additional file 14: Figure S12a, b), with structural morphology well preserved by this simple fructose-based clearing technique. Additionally, SeeDB-cleared samples were also highly compatible with rehydration and additional whole-mount immunostaining or with paraffin embedding, sectioning and 2D immunostaining (Additional file 14: Figure S12c). Collectively, these properties of optical transparency, morphology preservation, quality of whole-mount immunostaining and suitability for rehydration and 2D analyses, made SeeDB our preferred method for 3D fluorescence imaging of the mammary gland.

### Three-dimensional imaging of mammary tumours

**Fig. 6:**
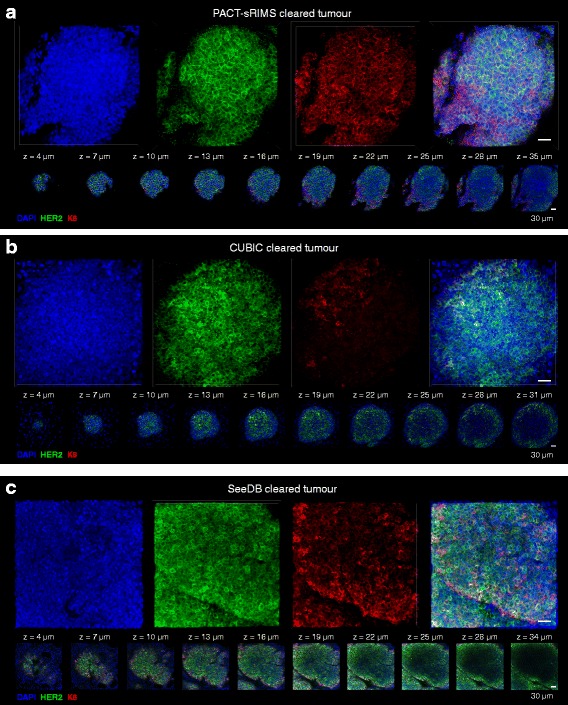
Three-dimensional confocal imaging of mouse mammary tumours cleared with the passive clarity technique (*PACT*)-sorbitol refractive index matching solution (*sRIMS*), clear unobstructed brain imaging cocktails (*CUBIC*) and see deep brain (*SeeDB*) methods. PACT-sRIMS cleared tumour tissue (**a**), CUBIC-cleared tumour tissue (**b**) and SeeDB-cleared tumour tissue (**c**). Images show maximum intensity projections of 4′,6-diamidino-2-phenylindole (*DAPI*) nuclear staining and human epidermal growth factor receptor 2 (*HER2*) and K8 immunostaining, with thin optical slices (1 μm) and their depth (*z* value) relative to the first image in the image sequence. These images are representative of at least two regions acquired. See additional file 18 for a high resolution version of these PDFs and http://rdcu.be/lT3Z for additional high resolution examples of these imaging techniques [38]

We also tested the performance of PACT-sRIMS, CUBIC and SeeDB clearing protocols on mouse mammary tumours derived from the syngeneic TUBO cell line [17]. All clearing protocols permitted high resolution imaging of HER2-expressing cells in mammary tumours at enhanced depths (Fig. 6 and Additional file 15: Movie 3). Similar to virgin and lactating tissue, K8 immunostaining was less intense in CUBIC-cleared mammary tumours (Fig. 6b), but may be improved by the second generation formulation (Reagent 1A). We observed that HER2, K8 and DAPI fluorescence intensity was reduced with increasing imaging depth in all protocols (Fig. 6). Whilst this may in part be attributable to a technical artefact, e.g. sub-optimal fixation or inadequate antibody penetration, it may also be a reflection of the inherent biology and heterogeneity of these tumour samples. Indeed, 2D immunohistochemical analysis revealed that the centre of many tumour lobules contained cleaved caspase-3-positive cells within areas of low E-cadherin staining (Additional file 16: Figure S13), highlighting the value of performing 2D analyses and 3D imaging in parallel when characterising complex and heterogeneous specimens.

**Fig. 7:**
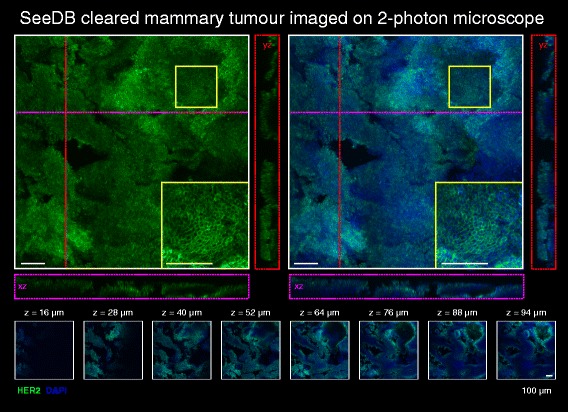
Imaging of see deep brain (*SeeDB*)-cleared mammary tumours using 2-photon excitation microscopy. Two-photon imaging of SeeDB-cleared human epidermal growth factor receptor 2 (*HER2*)-positive mammary tumours immunostained with HER2 (*green*) and 4′,6-diamidino-2-phenylindole (*DAPI*) nuclear staining (*blue*) and magnified view (*inset*). Orthagonal views show *XZ* (*purple line and box*) and *YZ* (*red line and box*) planes. Thin optical slices (2 μm) and their depth (*z* value) relative to the first image in the image sequence are also shown. See additional file 18 for a high resolution version of these PDFs and http://rdcu.be/lT3Z for additional high resolution examples of these imaging techniques [38]

Finally, we utilised two-photon excitation microscopy (TPEM) and light sheet fluorescence microscopy (LSFM) for rapid, large-scale imaging of normal and tumourigenic mammary tissue at improved depths and speeds (Fig. 7 and Additional file 17: Figure S14). TPEM uses longer wavelengths, thus allowing deeper penetration, less scattering of light and reduced out-of-focus photobleaching than laser scanning confocal microscopy [31]. Using this technique, we were able to image approx. 1.2 × 1.2 × 0.1 mm of SeeDB-cleared tumour tissue in less than 15 minutes per individual channel at high cellular resolution (Fig. 7). Deeper imaging was achievable (Fig. 7, orthogonal projections); however, as seen with confocal microscopy, HER2 immunostaining declined with increasing depth (Fig. 7, optical slices). Furthermore, using a home-built LSFM [27], with dual side illumination, we were able to image a volume of 0.8 × 0.8 × 1.5 mm in normal mammary tissue with CUBIC clearing in less than 5 min per individual channel (Additional file 17: Figure S14). These data demonstrate that SeeDB-cleared and CUBIC-cleared mammary tissue are also compatible with advanced imaging techniques.

## Discussion

Recent developments and refinements in whole-organ tissue clearing and 3D imaging techniques, such as LSFM, optical projection tomography (OPT) and X-ray tomography, have provided unprecedented optical access to intact mammalian tissues [32]. These protocols have been utilised for a range of biological applications, including neuronal circuit reconstruction [8–11, 15, 28, 33], characterisation of arterial wall structure [34] and single-cell lineage tracing in the embryonic heart [35]. In this technical report, we compared leading tissue clearing protocols for optical transparency, structural preservation and 3D fluorescent imaging in the intact mammary gland. This is the first study to employ tissue clearing protocols to examine mammary epithelial cells at single-cell resolution within their native stroma.

We first assessed these protocols for their ability to render opaque mammary tissue transparent. SeeDB and CUBIC clearing techniques achieved the highest degree of optical clarity in mammary tissue (Table 1). Due to the exceptional clarity achieved using the CUBIC protocol, we also investigated its suitability as an alternative to xylene delipidation for macroscopic imaging of mouse mammary gland whole mounts. CUBIC clearing combined with methyl green staining resulted in uniform, single-colour staining of mammary epithelial ducts and alveoli, and improved-contrast counterstaining for multicolour histochemical analysis. Importantly, CUBIC clearing was also compatible with whole-mount chromogenic immunostaining using HRP-DAB detection. Additionally, whole-mount-imaged tissue could be easily recovered and sectioned for subsequent 2D immunohistochemical analysis. For these reasons, we propose CUBIC clearing as a novel, superior clearing agent for mammary gland whole mounts.

**Table 1:** A comparison of selected clearing methods in the mammary gland

Method	Method overview	RI	Clearing capability	Duration^a^	Preservation		Whole mount IHC	Long-term storage	Rehydration and sectioning	Reference
					structure	FP				
Uncleared	No clearing protocol applied	−	−	1 day	Preserved	Preserved	Compatible	No	Possible^f^	−
3DISCO	Organic solvent-based	1.56	Strong	2 days	Compromised	Rapid loss	Difficult^c^	No	Not possible	[8]
PACT-RC	Aqueous solution-based (hydrogel embedding)	1.45	Weak	10 days	Preserved, mild expansion	Preserved	Compatible	Yes	Not possible	[11]
PACT-sRIMS	Aqueous solution-based (hydrogel embedding)	1.46	Weak	13 days	Preserved	Preserved	Compatible	No	Possible^f^	[11]
CUBIC	Aqueous solution-based (simple immersion)	1.48–9	Strong	5 days	Preserved, mild expansion	Some loss^b^	Semi-compatible^d^	No	Confirmed	[10]
SeeDB	Aqueous solution-based (simple immersion)	1.49	Moderate	5 days	Preserved, mild shrinkage	Preserved	Compatible	No^e^	Confirmed	[9]

^a^Duration from the time of tissue harvest (includes fixation time typically 6–16 h for mammary tissue). ^b^As previously reported [10, 39] and the observed requirement for slightly higher laser power for confocal imaging. This may be improved by using the second generation protocol (using Reagent 1A). ^c^Using the three-dimensional imaging of solvent-cleared organs combined with optimised whole-mount immunolabelling procedures iDISCO/three-dimensional imaging of solvent-cleared organs (3DISCO) method. The fluorescence signal is rapidly quenched using benzyl alcohol benzyl benzoate (BABB) and a specialised imaging chamber is required for dibenzyl ether (DBE). ^d^Some antibodies (i.e. E-cadherin, K8 and human epidermal growth factor receptor 2) do not perform as well as in other clearing protocols. ^e^Optimally imaged within 2 weeks but may be stored for several months. ^f^Likely to be compatible, but not tested in this study. *RI* refractive index, *FP* fluorescent protein, *IHC* immunohistochemical analysis, *PACT-RC* passive clarity technique-Rapiclear, *sRIMS* sorbitol refractive index matching solution, *CUBIC* clear unobstructed brain imaging cocktails, *SeeDB* see deep brain

A major limitation of early tissue-clearing techniques is pronounced sample volume changes, leading to cellular deformations [12, 28]. Here, we found that mammary tissue cleared with PACT-RC and CUBIC was associated with a moderate degree of sample expansion, as previously observed in brain tissue cleared using these techniques [10, 11]. Conversely, SeeDB was associated with minor shrinkage of mammary tissue (Table 1), while PACT-sRIMS had no effect on sample volume. Importantly, morphological deformations were not observed by confocal microscopy for any of the four main tissue clearing protocols assessed in this study. Thus, these small, predictable changes in linear sample volume are not likely to pose a major problem for the majority of studies in the mammary gland, provided the control/comparator mammary gland is also subjected to the same clearing protocol. Additionally, in contrast to mechanical dissection or enzymatic digestion, most optical tissue clearing protocols preserve both tissue and matrix architecture, and thus facilitate 3D imaging and analysis of epithelial-stromal interactions.

Whilst important biological information can be garnered from 3D imaging of near-surface structures in uncleared tissue [36, 37], the advantages presented by tissue clearing for the visualisation of expansive areas of mammary tissue are considerable, e.g. for comprehensive clonal analysis in lineage tracing studies [38] or mapping the cellular circuitry driving mammary gland development. Using tissue clearing protocols we were able to image genetically encoded fluorescent proteins and a range of immunolabelled lineage markers at vastly improved depths and at high cellular resolution in virgin, lactating and tumour tissue. Adoption of these protocols combined with volumetric imaging and published de-noising algorithms will greatly enhance our understanding of the structural organisation and development of the normal mammary gland, and how these processes are subverted in cancer. An evaluation of the performance of these protocols for diagnostic and experimental studies using clinical tumour biopsies is an aim for the future.

Although not evaluated in this study, it is expected that other antibodies, with appropriate optimisation, would be compatible with whole-mount immunostaining, tissue clearing and 3D imaging in the mammary gland. Thus, this technique has widespread applications in the broader fields of mammary gland biology and pathology. In particular, appropriate optimisation of the tissue fixation time is paramount for whole-mount immunostaining, which is not compatible with heat-induced epitope retrieval. Reduced performance of some antibodies was observed with CUBIC clearing in the mammary gland. This method relies on high concentrations of detergent (15% triton-X100) for clearing and, unlike the PACT protocol, does not entail prior hydrogel embedding to stabilise cellular structures, raising concerns that this protocol may be associated with some protein loss (Table 1) [10]. Indeed, this may explain the compromised fluorescence immunostaining of K8, E-cadherin and HER2 observed with the CUBIC clearing protocol in our study. To overcome this issue an updated CUBIC protocol has recently been developed (unpublished, see “Methods”) aimed at improving issues related to protein loss [39]. Alternatively, samples could be gel-embedded according to the CLARITY protocol [15] prior to CUBIC clearing; however, this has not been rigorously assessed in this or other [10] studies, and would require further optimisation for mammary tissue.

An assessment of the qualities of optical transparency, structural preservation, imaging depth, immunostaining, compatibility with downstream analyses, cost and safety (Table 1), placed SeeDB as our method of choice for tissue clearing and volumetric imaging of mammary tissue using standard confocal and advanced fluorescence imaging techniques. The deep tissue imaging of normal and pathological mammary tissue will greatly improve our understanding of this architecturally complex and heterogeneous organ.

## Conclusions

This technical report compared the strengths and limitations of a range of whole-organ tissue clearing protocols for optical transparency and 3D imaging in the mouse mammary gland. Notably, the methods examined here are open source protocols, which utilise reagents that are both widely available and affordable to most laboratories. Additionally, whilst these protocols are compatible with advanced imaging techniques, they can also be paired with standard confocal microscopy and open source analysis platforms for universal use. We hope that this publication sheds light on the methods available for optical tissue clearing of the mouse mammary gland, and encourages other researchers to perform their mammary tissue imaging in 3D, with all architectural information preserved.

## Additional files

Additional file 1: Figure S1. No primary antibody controls of CUBIC cleared tissue (*top panel*) and SeeDB cleared tissue (*bottom panel*). See Additional file 18 for a high resolution version of these PDFs. (PDF 243 mb)
Additional file 2: Figure S2. 3DISCO clearing and 3D imaging of virgin and lactating mouse mammary tissue. **a** 3DISCO tissue clearing and immunostaining protocol and timeline. **b** Transmission images of 3DISCO cleared tissue. **c** Volume changes caused by 3DISCO-based clearing of virgin and lactating mammary tissue. Values are representative of measurements from three tissue pieces at each developmental timepoint. **d** 3D confocal imaging of 3DISCO-cleared virgin and lactating mammary glands immunostained with the basal cellb marker SMA. These images are representative of images from more than two mice. See Additional file 18 for a high resolution version of these PDFs. (PDF 8 mb)
Additional file 3: Figure S3. PACT-RC clearing and 3D imaging of virgin and lactating mouse mammary tissue. **a** PACT-RC tissue clearing and immunostaining protocol and timeline. **b** The optimal (*i*) and PACT-RC (*ii*) imaging configuration for confocal microscopy. For optimal illumination, samples are squeezed between two glass coverslips and are easily flipped or re-positioned for imaging. In contrast, for PACT-RC-cleared samples, samples are mounted using an iSpacer chamber and are difficult to adjust or reposition for optimal illumination, thus the working distance becomes the limiting factor in image acquisition. To overcome this, sample thickness must be closely matched to the thickness of the iSpacer chamber, specialised imaging objectives need to be used or different RI-matching solutions are needed. **c** 3D confocal imaging of PACT-RC-cleared virgin and lactating mammary glands immunostained with the basal cell marker SMA and stained with the nuclear stain DAPI. These images are representative of images from at least two mice. See Additional file 18 for a high resolution version of these PDFs. (PDF 124 mb)
Additional file 4: Figure S4. 3D imaging of uncleared virgin and lactating mouse mammary tissue. **a** Immunostaining protocol and timeline, without a tissue clearing step. 3D confocal imaging of uncleared virgin (**b**) and lactating (**c**) mammary glands immunostained with basal cell marker SMA and the luminal cell marker E-cadherin. Main image shows the maximum intensity projection of the entire image sequence, with thin optical slices (1 μm) and their depth (*z* value) relative to the first image in the image sequence. Visible structures were located very close to the surface of the tissue. See Additional file 18 for a high resolution version of these PDFs. (PDF 176 mb)
Additional file 5: Figure S5. De-noising of 3D image sequences. DAPI staining (*top panel*) and E-cadherin immunostaining (*bottom panel*) in lactating mammary tissue before and after a de-noising algorithm was applied to minimise Poisson-Gaussian noise in the 3D image stacks. De-noising does not greatly alter the outward appearance of the image sequence, but rather aids downstream computer-assisted analyses. See Additional file 18 for a high resolution version of these PDFs. (PDF 4 mb)
Additional file 6: Figure S6. Additional 3D confocal images of PACT-sRIMS-cleared mammary glands, related to Fig. 2. *BV* blood vessel (SMA-expressing). See Additional file 18 for a high resolution version of these PDFs. (PDF 13 mb)
Additional file 7: Movie 1. Three-dimensional rendering of SMA immunostaining (*red*) and DAPI nuclear staining (*blue*) in CUBIC-cleared lactating mammary tissue. Movie shows a layer of basket-like basal cells surrounding each alveolus with single cell resolution. (AVI 20400 kb)
Additional file 8: Figure S7. Additional 3D confocal images of CUBIC-cleared mammary glands, related to Fig. 3. *Arrowhead* shows non-specific intraluminal staining occasionally observed with CUBIC clearing, which may be improved with more rigorous washing following immersion in Reagent 1. See Additional file 18 for a high resolution version of these PDFs. (PDF 23 mb)
Additional file 9: Figure S8. Three-dimensional confocal images of mammary glands cleared using a modified CUBIC clearing protocol (Reagent 1A) to minimise protein loss. Images show the maximum intensity projection of the entire image sequence. *Bottom right panel* shows depth-coding of SMA-expressing myoepithelial cells in lactating mammary tissue; images in an image stack are assigned a colour based on their relative depth. *Arrowhead* shows non-specific intraluminal staining occasionally observed with modified CUBIC clearing, which may be improved with further washing. These images are representative of images from two mice. *BV* blood vessel (SMA-expressing). See Additional file 18 for a high resolution version of these PDFs. (PDF 12 mb)
Additional file 10: Figure S9. Compatibility of CUBIC clearing with genetically encoded FPs and recovery for histological sections. **a** CUBIC clearing of tissue from R26-Confetti mice with reporter expression (nuclear GFP, cytosolic YFP and cytosolic RFP) induced at very low, sporadic levels. Membranous CFP was not observed with any clearing protocol and may be technical; however, this FP is also reportedly underrepresented in mammary tissue from R26-Confetti mice. **b** Tissue from lactating R26-Tdtomato mice induced at very high levels. **c** CUBIC-cleared tissue was rehydrated in PBS prior to standard processing, paraffin embedding and sectioning. Immunostaining for E-cadherin (luminal) and SMA (basal) markers confirm the compatibility of CUBIC clearing with tissue recovery and immunostaining. See Additional file 18 for a high resolution version of these PDFs. (PDF 126 mb)
Additional file 11: Figure S10. Compatibility of CUBIC clearing with carmine and haematoxylin whole-mount staining. Carmine staining (*top*) shows light pink, non-uniform staining in virgin and lactating tissue. Haematoxylin staining (*bottom*) was an intense blue colour in both ducts and stroma. See Additional file 18 for a high resolution version of these PDFs. (PDF 30 mb)
Additional file 12: Movie 2. Three-dimensional rendering of SMA (*green*) and K8 (*red*) immunostaining in SeeDB-cleared virgin mammary tissue. Movie shows luminal and basal cell layers with single cell resolution. (AVI 32300 kb)
Additional file 13: Figure S11. Additional 3D confocal images of SeeDB-cleared mammary glands, related to Fig. 5. See Additional file 18 for a high resolution version of these PDFs. (PDF 10 mb)
Additional file 14: Figure S12. Compatibility of SeeDB clearing with genetically encoded FPs and recovery for histological sections. **a** SeeDB clearing of tissue from virgin R26-Confetti mice with reporter expression (nuclear GFP, cytosolic YFP and cytosolic RFP) induced at very low, sporadic levels. Membranous CFP was not observed with any clearing protocol and may be technical; however, this FP is also reportedly underrepresented in mammary tissue from R26-Confetti mice. **b** Tissue from lactating R26-Tdtomato mice induced at very high levels. **c** SeeDB-cleared tissue was rehydrated in PBS prior to standard processing, paraffin embedding and sectioning. Immunostaining for K8 (luminal) and SMA (basal) markers confirm the compatibility of SeeDB clearing with tissue recovery and immunostaining. See Additional file 18 for a high resolution version of these PDFs. (PDF 154 mb)
Additional file 15: Movie 3. Three-dimensional rendering of HER2 (*red*) and K8 (*green*) immunostaining with DAPI nuclear staining (*blue*) in SeeDB-cleared mammary tumours. Movie shows optical slices and the single cell resolution, allowing visualisation of the 3D structure of the tumour tissue. (AVI 45300 kb)
Additional file 16: Figure S13. Two dimensional immunohistochemical analysis of tumour fragments. K8 and HER2 (**a**) and E-cadherin and cleaved caspase-3 (CC3) (**b**) immunostaining on formalin-fixed paraffin embedded tissue sections from mouse mammary tumours. CC3-positive cells can be observed close to the tumour boundary and in the centre of the tumour fragment, and thus, are not simply an artefact of sub-optimal tissue fixation. *Dotted line* in *left panel* shows the tissue boundary. DAPI nuclear staining (*blue*). See Additional file 18 for a high resolution version of these PDFs. (PDF 26 mb)
Additional file 17: Figure S14. LSFM imaging in the mammary gland. SMA immunostaining and CUBIC clearing of mammary tissue from (**a**) early gestation (total depth 800 μm) and (**b**) virgin (total depth 500 μm) mammary tissue. *BV* blood vessel (SMA-expressing). See Additional file 18 for a high resolution version of these PDFs. (PDF 9 mb)
Additional file 18: All figures in high resolution. (ZIP 127 MB)

## Abbreviations

2D: two-dimensional
3D: three-dimensional
3DISCO: three-dimensional imaging of solvent-cleared organs
BABB: benzyl alcohol benzyl benzoate
BSA: bovine serum albumin
BV: blood vessel
CUBIC: clear unobstructed brain imaging cocktails
DAB: 3,3-diaminobenzidine
DAPI: 4′,6-diamidino-2-phenylindole
DBE: dibenzyl ether
DCM: dichloromethane
E-CAD: E-cadherin
FP: fluorescent protein
HER2: human epidermal growth factor receptor 2
HRP: horseradish peroxidase
iDISCO: three-dimensional imaging of solvent-cleared organs combined with optimised whole-mount immunolabelling procedures
IHC: immunohistochemistry
K5: keratin 5
K8: keratin 8
LSFM: light sheet fluorescence microscopy
NBF: neutral buffered formalin
OPT: optical projection tomography
PACT: passive clarity technique
PBS: phosphate-buffered saline
RC: Rapiclear
RI: refractive index
SeeDB: see deep brain
SMA: smooth muscle actin
sRIMS: sorbitol refractive index matching solution
THF: tetrahydrofuran
TPEM: two-photon excitation microscopy

## References

[1] Macias H, Hinck L. Mammary gland development. Wiley Interdiscip Rev Dev Biol. 2012;1:533–57. 10.1002/wdev.35 PMC3404495 22844349

[2] Rios AC, Fu NY, Lindeman GJ, Visvader JE (2014). In situ identification of bipotent stem cells in the mammary gland. Nature.

[3] Wuidart A, Ousset M, Rulands S, Simons B, Van Keymeulen A, Blanpain C (2016). Quantitative lineage tracing strategies to resolve multipotency in tissue-specific stem cells. Genes Dev.

[4] Wang D, Cai C, Dong X, Yu QC, Zhang X-O, Yang L (2015). Identification of multipotent mammary stem cells by protein C receptor expression. Nature.

[5] Rios AC, Fu NY, Jamieson PR, Pal B, Whitehead L, Nicholas KR (2016). Essential role for a novel population of binucleated mammary epithelial cells in lactation. Nat Commun.

[6] Spalteholz W. Uber das Durchsichtigmachen von menschlichen und tierischen Praparaten. Leipzig; 1914.

[7] Tainaka K, Kuno A, Kubota S, Murakami T, Ueda H (2016). Chemical principles in tissue clearing and staining protocols for whole-body cell profiling. Annu Rev Cell Dev Biol.

[8] Erturk A, Becker K, Jahrling N, Mauch CP, Hojer CD, Egen JG (2012). Three-dimensional imaging of solvent-cleared organs using 3DISCO. Nat Protoc.

[9] Ke M-T, Fujimoto S, Imai T (2013). SeeDB: a simple and morphology-preserving optical clearing agent for neuronal circuit reconstruction. Nat Neurosci.

[10] Susaki EA, Tainaka K, Perrin D, Kishino F, Tawara T, Watanabe TM (2014). Whole-brain imaging with single-cell resolution using chemical cocktails and computational analysis. Cell.

[11] Yang B, Treweek JB, Kulkarni RP, Deverman BE, Chen CK, Lubeck E (2014). Single-cell phenotyping within transparent intact tissue through whole-body clearing. Cell.

[12] Becker K, Jährling N, Saghafi S, Weiler R, Dodt HU. Chemical clearing and dehydration of GFP expressing mouse brains. PLoS One. 2012;7:e33916. 10.1371/journal.pone.0033916 PMC3316521 22479475

[13] Dodt H-U, Leischner U, Schierloh A, Jährling N, Mauch CP, Deininger K (2007). Ultramicroscopy: three-dimensional visualization of neuronal networks in the whole mouse brain. Nat Methods.

[14] Renier N, Wu Z, Simon DJ, Yang J, Ariel P, Tessier-Lavigne M (2014). iDISCO: a simple, rapid method to immunolabel large tissue samples for volume imaging. Cell.

[15] Chung K, Deisseroth K (2013). CLARITY for mapping the nervous system. Nat Methods.

[16] Tainaka K, Kubota SI, Suyama TQ, Susaki EA, Perrin D, Ukai-Tadenuma M (2014). Whole-body imaging with single-cell resolution by tissue decolorization. Cell.

[17] Rovero S, Amici A, Di Carlo E, Bei R, Nanni P, Quaglino E (2000). DNA vaccination against rat her-2/Neu p185 more effectively inhibits carcinogenesis than transplantable carcinomas in transgenic BALB/c mice. J Immunol.

[18] Marx V (2014). Microscopy: seeing through tissue. Nat Methods.

[19] McCutcheon SC, Jones K, Cumming SA, Kemp R, Ireland-Zecchini H, Saunders JC (2010). Characterization of a heat resistant beta-glucosidase as a new reporter in cells and mice. BMC Biol.

[20] Manousiouthakis E, Mendez M, Garner MC, Exertier P, Makita T (2014). Venous endothelin guides sympathetic innervation of the developing mouse heart. Nat Commun.

[21] Dills WL. Protein fructosylation: fructose and the Maillard reaction. Am J Clin Nutr. 1993;58:779S-87S. 10.1093/ajcn/58.5.779S 8213610

[22] Sargeant TJ, Lloyd-Lewis B, Resemann HK, Ramos-Montoya A, Skepper J, Watson CJ (2014). Stat3 controls cell death during mammary gland involution by regulating uptake of milk fat globules and lysosomal membrane permeabilization. Nat Cell Biol.

[23] Schindelin J, Arganda-Carreras I, Frise E, Kaynig V, Longair M, Pietzsch T (2012). Fiji: an open source platform for biological image analysis. Nat Methods.

[24] Linkert M, Rueden CT, Allan C, Burel JM, Moore W, Patterson A, et al. Metadata matters: access to image data in the real world. J Cell Biol. 2010;189:777–82. 10.1083/jcb.201004104 PMC2878938 20513764

[25] Boulanger J, Kervrann C, Bouthemy P, Elbau P, Sibarita J-B, Salamero J (2010). Patch-based nonlocal functional for denoising fluorescence microscopy image sequences. IEEE Trans Med Imaging.

[26] Preibisch S, Saalfeld S, Tomancak P (2009). Globally optimal stitching of tiled 3D microscopic image acquisitions. Bioinformatics.

[27] Pitrone P, Schindelin J, Stuyvenberg L, Preibisch S, Weber M, Eliceiri K (2013). OpenSPIM: an open-access light-sheet microscopy platform. Nat Methods.

[28] Hama H, Kurokawa H, Kawano H, Ando R, Shimogori T, Noda H (2011). Scale: a chemical approach for fluorescence imaging and reconstruction of transparent mouse brain. Nat Neurosci.

[29] Plante I, Stewart MKG, Laird DW. Evaluation of mammary gland development and function in mouse models. J Vis Exp. 2011;53:2–6. 10.3791/2828 PMC3196158 21808224

[30] van Amerongen R. Lineage tracing in the mammary gland using Cre/lox technology and fluorescent reporter alleles. Methods Mol Biol. 2015;1293:187-211. 10.1007/978-1-4939-2519-3_11 26040689

[31] Drobizhev M, Makarov NS, Tillo SE, Hughes TE, Rebane A (2011). Two-photon absorption properties of fluorescent proteins. Nat Methods.

[32] Shearer T, Bradley R, Hidalgo-Bastida A, Sherratt M, Cartmell S (2016). Three-dimensional visualisation of soft biological structures by X-ray computed micro-tomography. J Cell Sci.

[33] Schmitt O, Modersitzki J, Heldmann S, Wirtz S, Fischer B (2007). Image registration of sectioned brains. Int J Comput Vis.

[34] Walton LA, Bradley RS, Withers PJ, Newton VL, Watson REB, Austin C (2015). Morphological characterisation of unstained and intact tissue micro-architecture by x-ray computed micro- and nano-tomography. Sci Rep.

[35] Li J, Miao L, Shieh D, Spiotto E, Li J, Zhou B (2016). Single-cell lineage tracing reveals that oriented cell division contributes to trabecular morphogenesis and regional specification. Cell Rep.

[36] Davis FM, Janoshazi A, Janardhan KS, Steinckwich N, D’Agostin DM, Petranka JG (2015). Essential role of Orai1 store-operated calcium channels in lactation. Proc Natl Acad Sci U S A.

[37] Raymond K, Cagnet S, Kreft M, Janssen H, Sonnenberg A, Glukhova MA (2011). Control of mammary myoepithelial cell contractile function by α3β1 integrin signalling. EMBO J.

[38] Davis, Lloyd-Lewis et al. Single-cell lineage tracing in the mammary gland reveals stochastic clonal dispersion of stem/progenitor cell progeny. Nature Communications. 2016. doi:10.1038/ncomms13053. 10.1038/ncomms13053 PMC5093309 27779190

[39] Susaki EA, Ueda HR (2016). Whole-body and whole-organ clearing and imaging techniques with single-cell resolution: toward organism-level systems biology in mammals. Cell Chem Biol.

