# Supplementary figures and images for: Imaging the mammary gland and mammary tumours in 3D: optical tissue clearing and immunofluorescence methods
Source: Breast Cancer Res. 2016 Dec 13;18:127. doi: 10.1186/s13058-016-0754-9 (PMC5155399; doi:10.1186/s13058-016-0754-9)

**a** 3DISCO/iDISCO clearing and immunostaining protocol

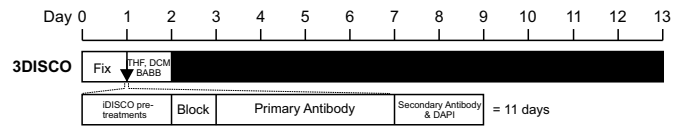

**b**

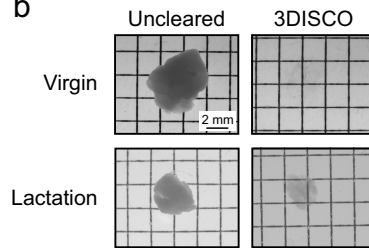

**c**

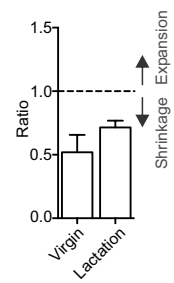

**d**

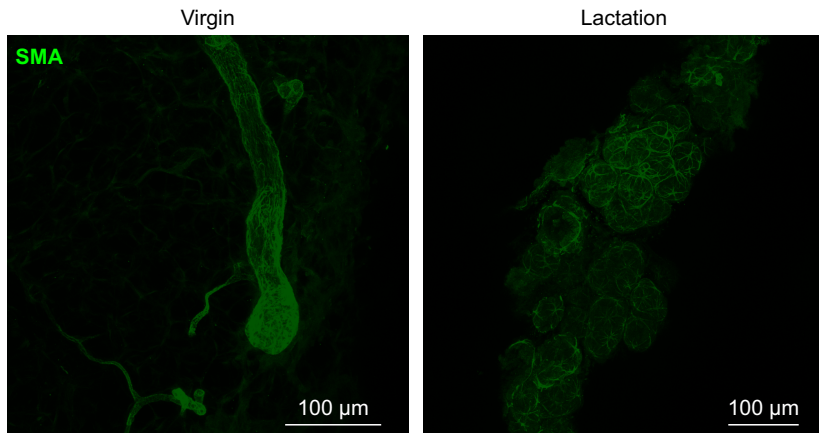

Supplement: Additional file 2: Figure S2. — 3DISCO clearing and 3D imaging of virgin and lactating mouse mammary tissue. a 3DISCO tissue clearing and immunostaining protocol and timeline. b Transmission images of 3DISCO cleared tissue. c Volume changes caused by 3DISCO-based clearing of virgin and lactating mammary tissue. Values are representative of measurements from three tissue pieces at each developmental timepoint. d 3D confocal imaging of 3DISCO-cleared virgin and lactating mammary glands immunostained with the basal cellb marker SMA. These images are representative of images from more than two mice. See Additional file 18 for a high resolution version of these PDFs. (PDF 8 mb) [file 13058_2016_754_MOESM2_ESM.pdf]

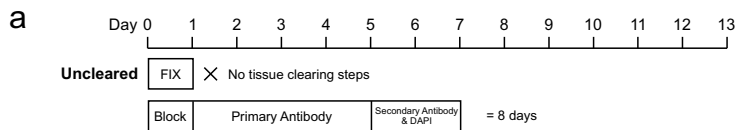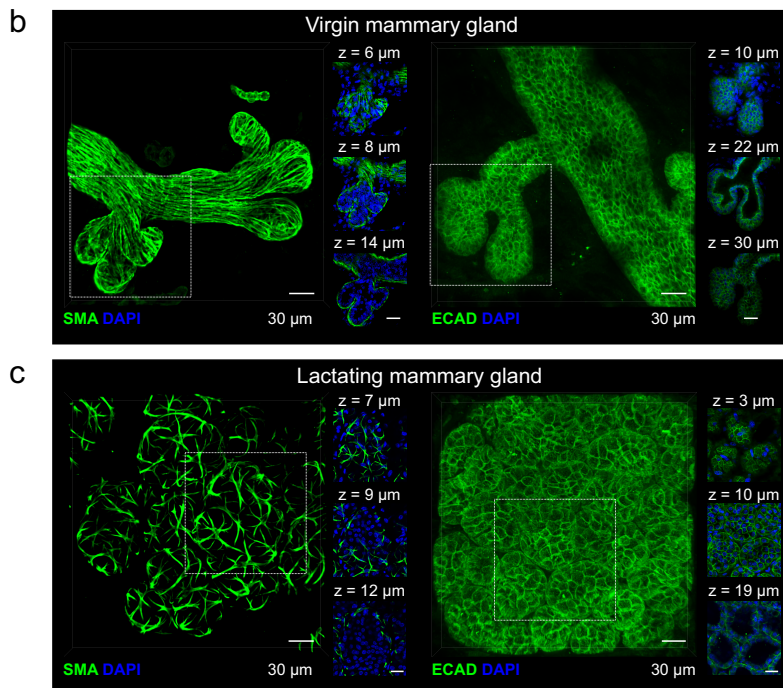

Supplement: Additional file 4: Figure S4. — 3D imaging of uncleared virgin and lactating mouse mammary tissue. a Immunostaining protocol and timeline, without a tissue clearing step. 3D confocal imaging of uncleared virgin (b) and lactating (c) mammary glands immunostained with basal cell marker SMA and the luminal cell marker E-cadherin. Main image shows the maximum intensity projection of the entire image sequence, with thin optical slices (1 μm) and their depth (z value) relative to the first image in the image sequence. Visible structures were located very close to the surface of the tissue. See Additional file 18 for a high resolution version of these PDFs. (PDF 176 mb) [file 13058_2016_754_MOESM4_ESM.pdf]

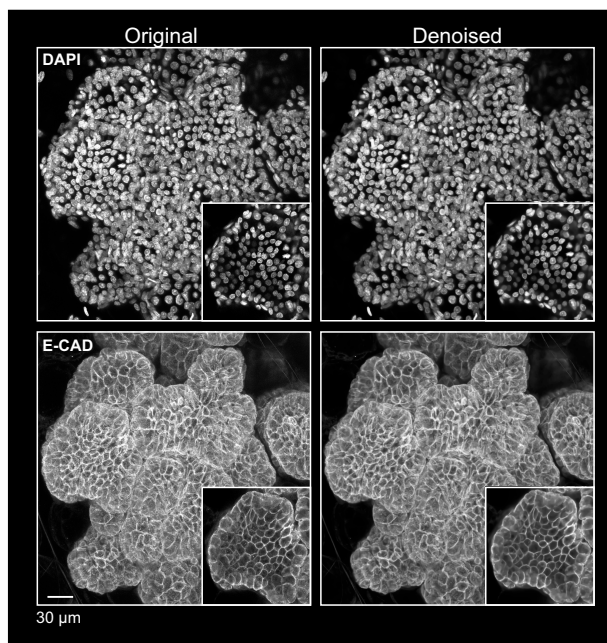

Supplement: Additional file 5: Figure S5. — De-noising of 3D image sequences. DAPI staining (top panel) and E-cadherin immunostaining (bottom panel) in lactating mammary tissue before and after a de-noising algorithm was applied to minimise Poisson-Gaussian noise in the 3D image stacks. De-noising does not greatly alter the outward appearance of the image sequence, but rather aids downstream computer-assisted analyses. See Additional file 18 for a high resolution version of these PDFs. (PDF 4 mb) [file 13058_2016_754_MOESM5_ESM.pdf]

Further examples of sRIMS clearing in the mammary gland

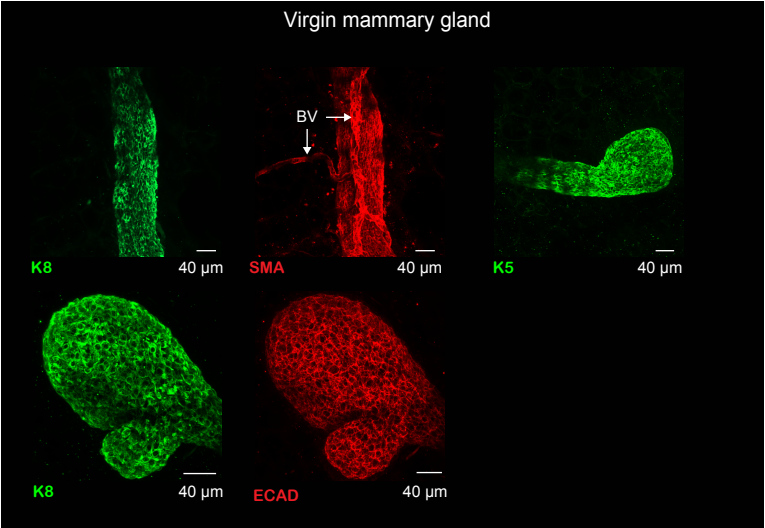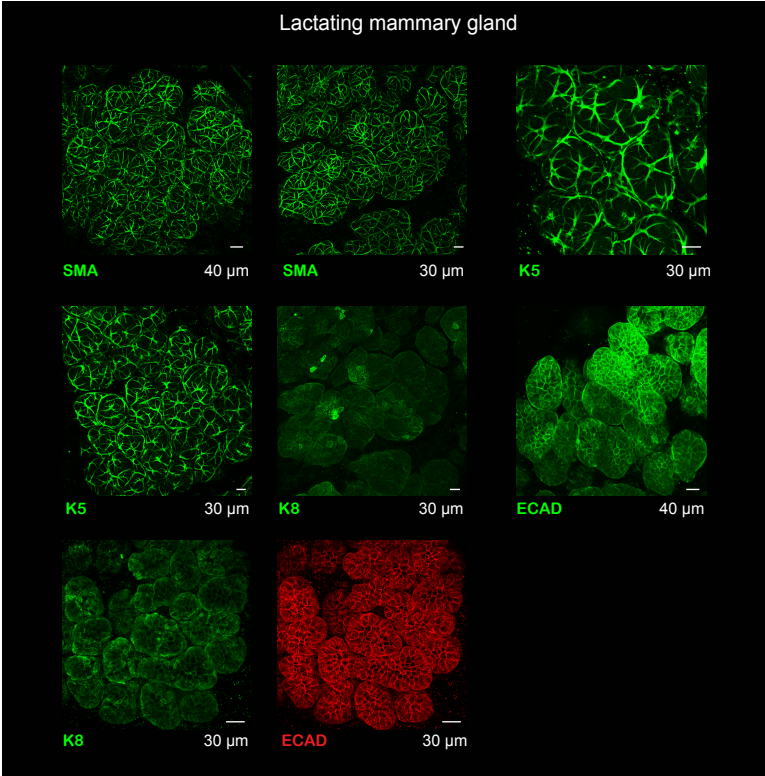

Supplement: Additional file 6: Figure S6. — Additional 3D confocal images of PACT-sRIMS-cleared mammary glands, related to Fig. 2. BV blood vessel (SMA-expressing). See Additional file 18 for a high resolution version of these PDFs. (PDF 13 mb) [file 13058_2016_754_MOESM6_ESM.pdf]

Modified CUBIC clearing in the mammary gland - Reagent 1A

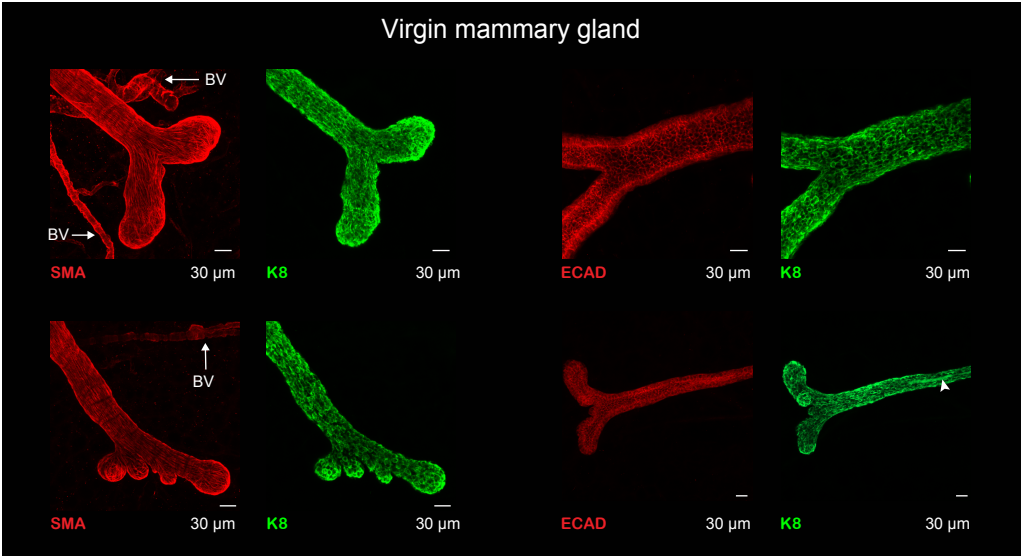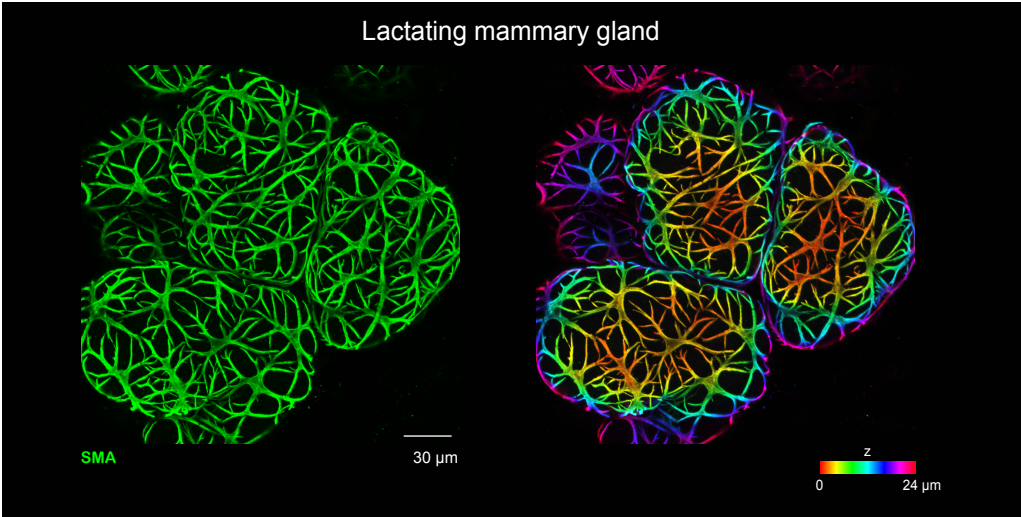

Supplement: Additional file 9: Figure S8. — Three-dimensional confocal images of mammary glands cleared using a modified CUBIC clearing protocol (Reagent 1A) to minimise protein loss. Images show the maximum intensity projection of the entire image sequence. Bottom right panel shows depth-coding of SMA-expressing myoepithelial cells in lactating mammary tissue; images in an image stack are assigned a colour based on their relative depth. Arrowhead shows non-specific intraluminal staining occasionally observed with modified CUBIC clearing, which may be improved with further washing. These images are representative of images from two mice. BV blood vessel (SMA-expressing). See Additional file 18 for a high resolution version of these PDFs. (PDF 12 mb) [file 13058_2016_754_MOESM9_ESM.pdf]

a

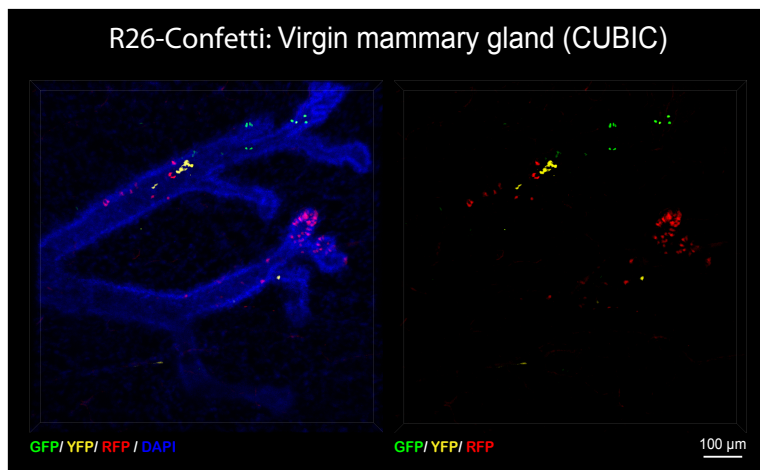

b

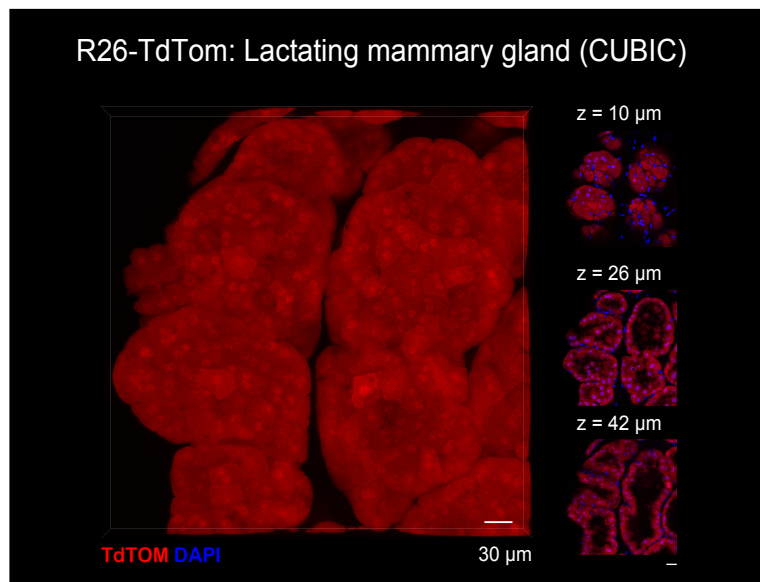

c

Histological sections from CUBIC-recovered tissue

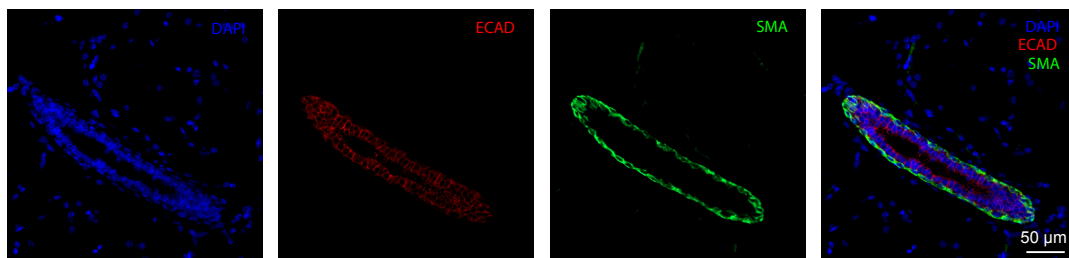

Supplement: Additional file 10: Figure S9. — Compatibility of CUBIC clearing with genetically encoded FPs and recovery for histological sections. a CUBIC clearing of tissue from R26-Confetti mice with reporter expression (nuclear GFP, cytosolic YFP and cytosolic RFP) induced at very low, sporadic levels. Membranous CFP was not observed with any clearing protocol and may be technical; however, this FP is also reportedly underrepresented in mammary tissue from R26-Confetti mice. b Tissue from lactating R26-Tdtomato mice induced at very high levels. c CUBIC-cleared tissue was rehydrated in PBS prior to standard processing, paraffin embedding and sectioning. Immunostaining for E-cadherin (luminal) and SMA (basal) markers confirm the compatibility of CUBIC clearing with tissue recovery and immunostaining. See Additional file 18 for a high resolution version of these PDFs. (PDF 126 mb) [file 13058_2016_754_MOESM10_ESM.pdf]

a

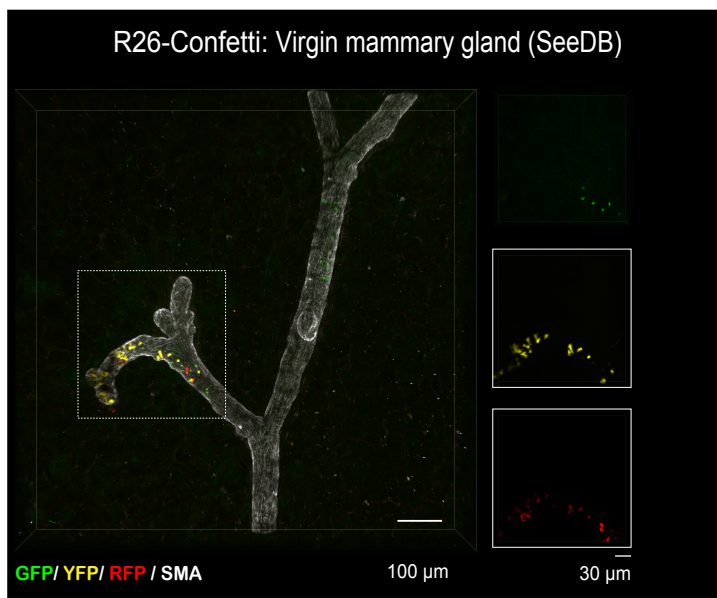

b

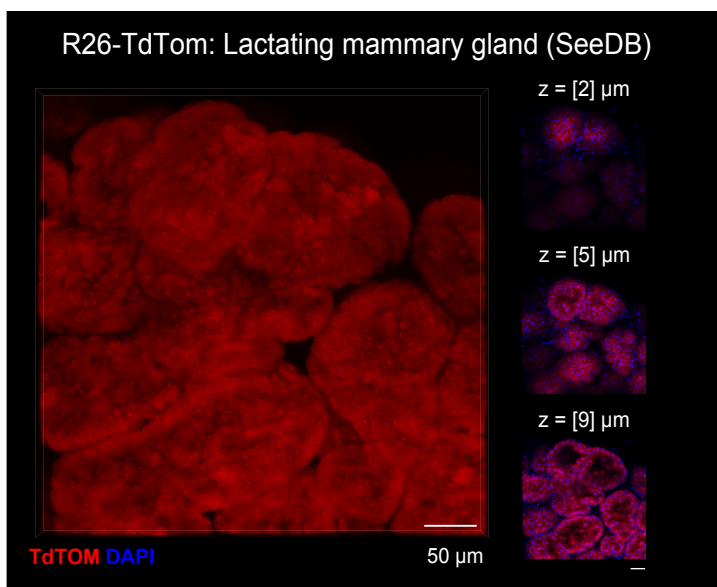

c

Histological sections from SeeDB-recovered tissue

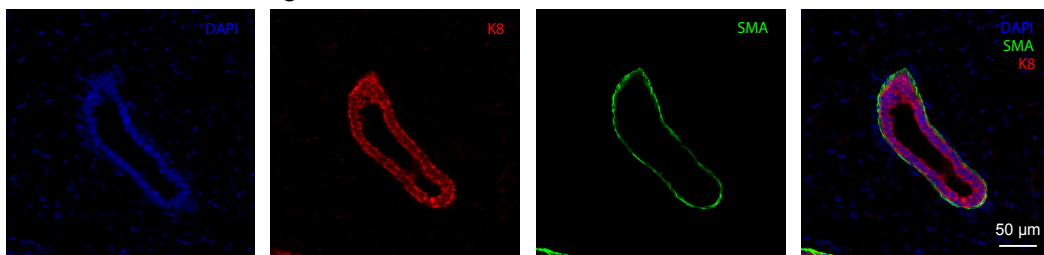

Supplement: Additional file 14: Figure S12. — Compatibility of SeeDB clearing with genetically encoded FPs and recovery for histological sections. a SeeDB clearing of tissue from virgin R26-Confetti mice with reporter expression (nuclear GFP, cytosolic YFP and cytosolic RFP) induced at very low, sporadic levels. Membranous CFP was not observed with any clearing protocol and may be technical; however, this FP is also reportedly underrepresented in mammary tissue from R26-Confetti mice. b Tissue from lactating R26-Tdtomato mice induced at very high levels. c SeeDB-cleared tissue was rehydrated in PBS prior to standard processing, paraffin embedding and sectioning. Immunostaining for K8 (luminal) and SMA (basal) markers confirm the compatibility of SeeDB clearing with tissue recovery and immunostaining. See Additional file 18 for a high resolution version of these PDFs. (PDF 154 mb) [file 13058_2016_754_MOESM14_ESM.pdf]

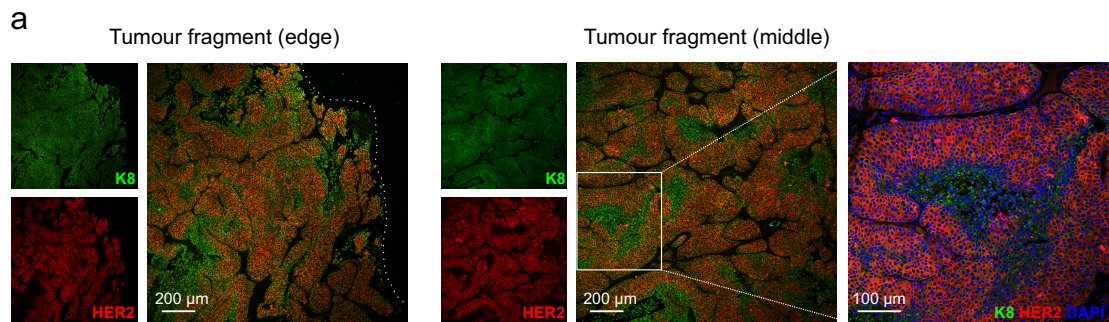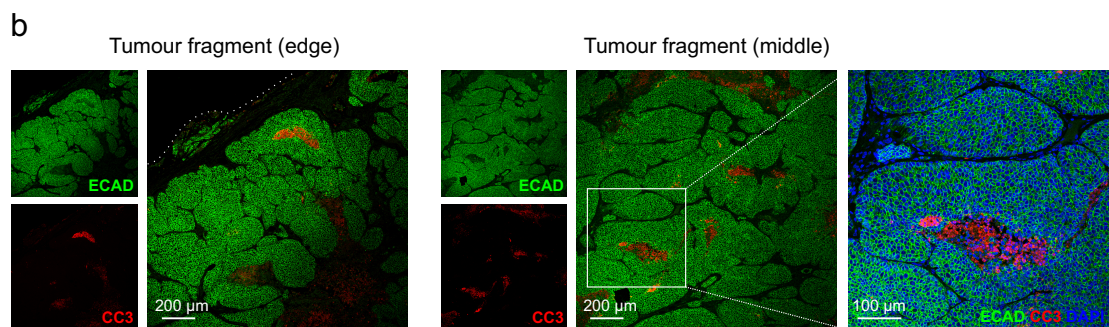

Supplement: Additional file 16: Figure S13. — Two dimensional immunohistochemical analysis of tumour fragments. K8 and HER2 (a) and E-cadherin and cleaved caspase-3 (CC3) (b) immunostaining on formalin-fixed paraffin embedded tissue sections from mouse mammary tumours. CC3-positive cells can be observed close to the tumour boundary and in the centre of the tumour fragment, and thus, are not simply an artefact of sub-optimal tissue fixation. Dotted line in left panel shows the tissue boundary. DAPI nuclear staining (blue). See Additional file 18 for a high resolution version of these PDFs. (PDF 26 mb) [file 13058_2016_754_MOESM16_ESM.pdf]

# CUBIC cleared mammary gland imaged using LSMF

a

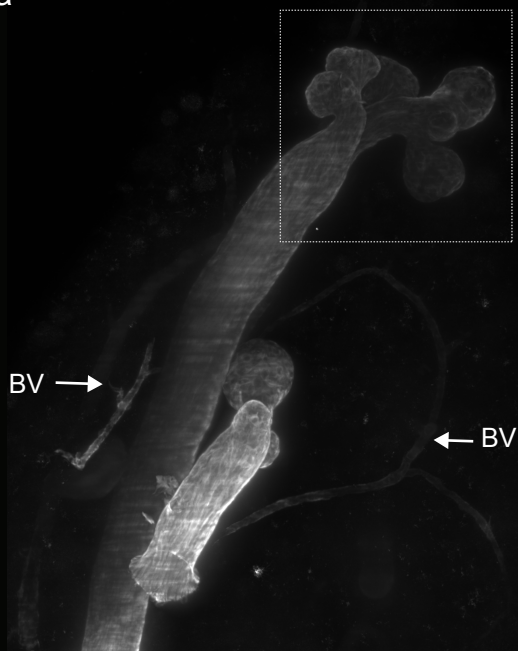

SMA

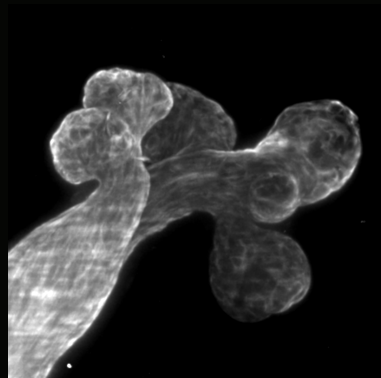

SMA

b

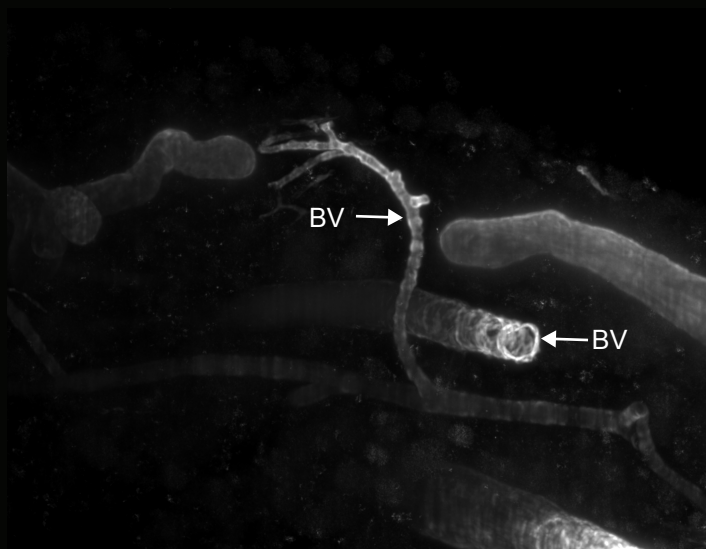

SMA

Supplement: Additional file 17: Figure S14. — LSFM imaging in the mammary gland. SMA immunostaining and CUBIC clearing of mammary tissue from (a) early gestation (total depth 800 μm) and (b) virgin (total depth 500 μm) mammary tissue. BV blood vessel (SMA-expressing). See Additional file 18 for a high resolution version of these PDFs. (PDF 9 mb) [file 13058_2016_754_MOESM17_ESM.pdf]

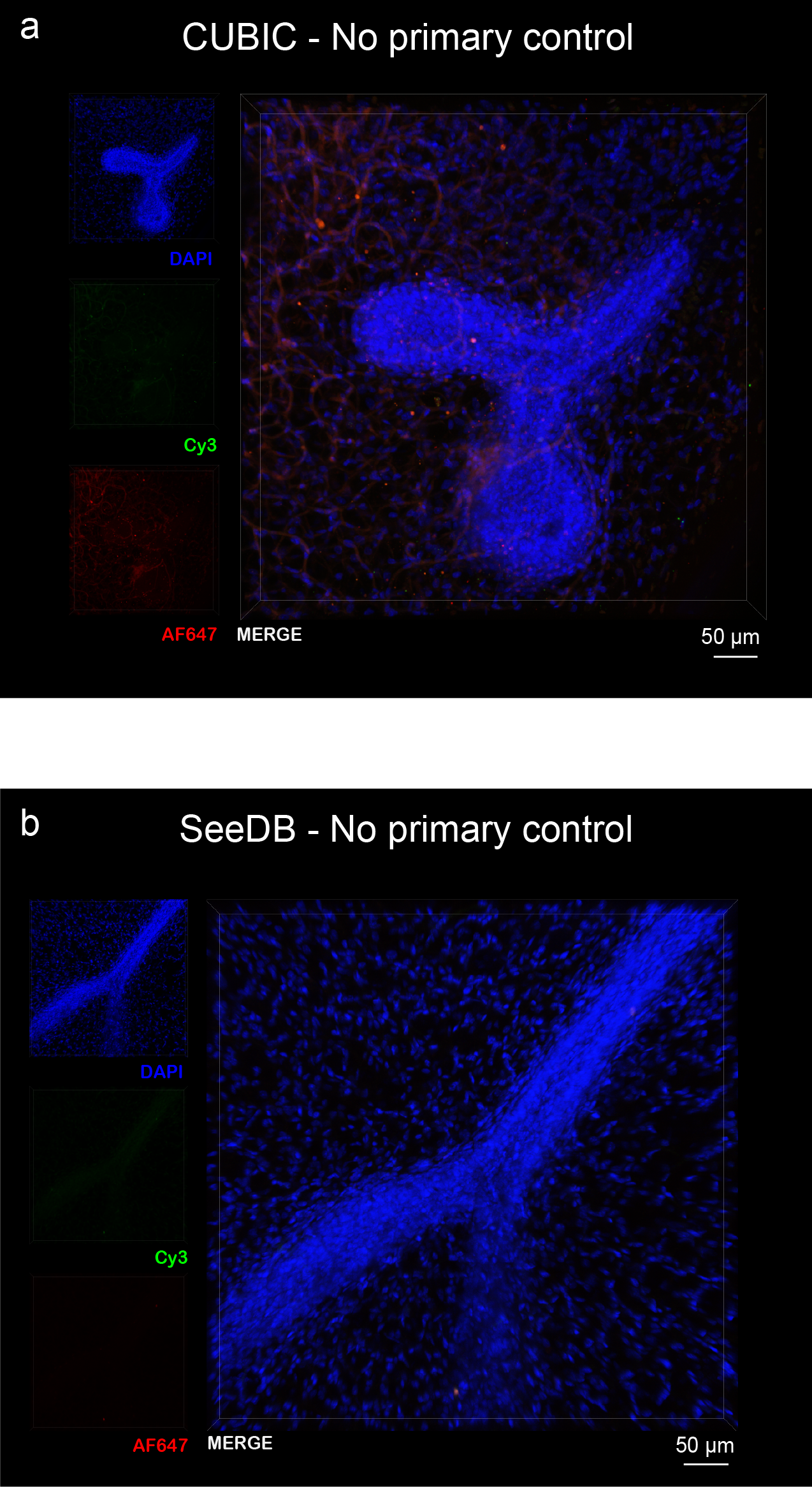

Supplement: Additional file 18: — All figures in high resolution. (ZIP 127 MB) [file 13058_2016_754_MOESM18_ESM.zip › Final final PNG for online links/Additional File 1 Fig S1.png]

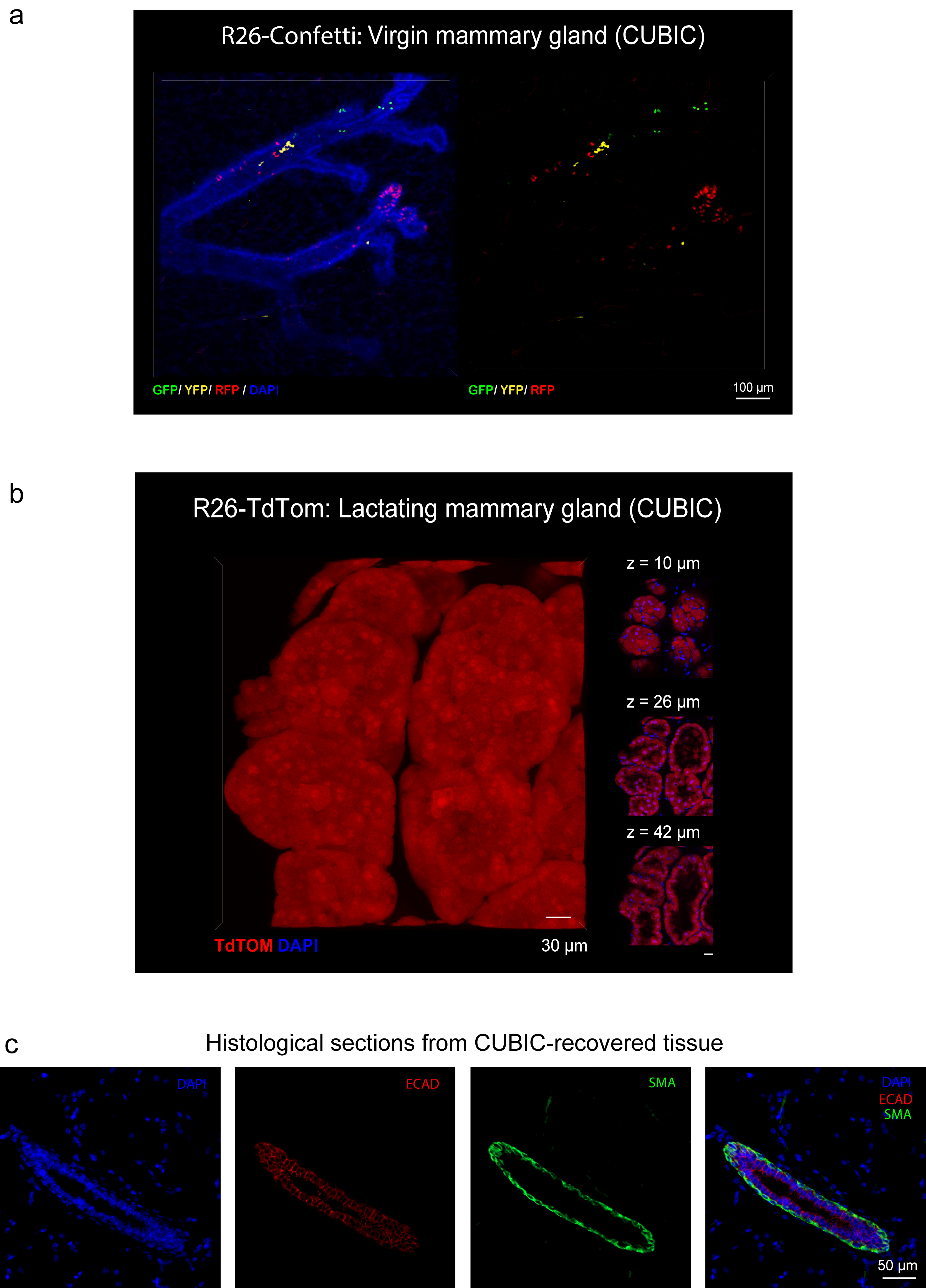

Supplement: Additional file 18: — All figures in high resolution. (ZIP 127 MB) [file 13058_2016_754_MOESM18_ESM.zip › Final final PNG for online links/Additional File 10 Fig S9.png]

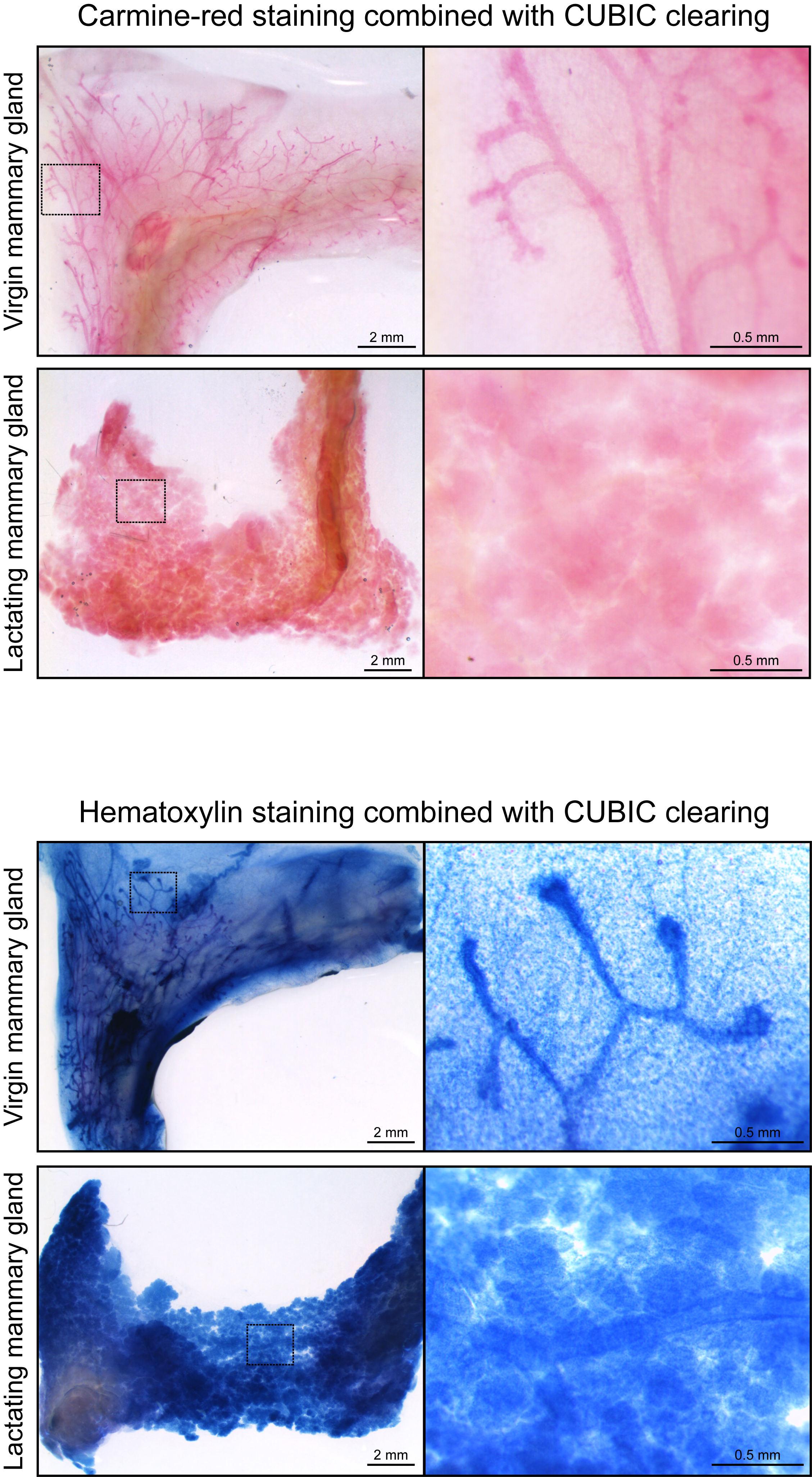

Supplement: Additional file 18: — All figures in high resolution. (ZIP 127 MB) [file 13058_2016_754_MOESM18_ESM.zip › Final final PNG for online links/Additional File 11 Fig S10.png]

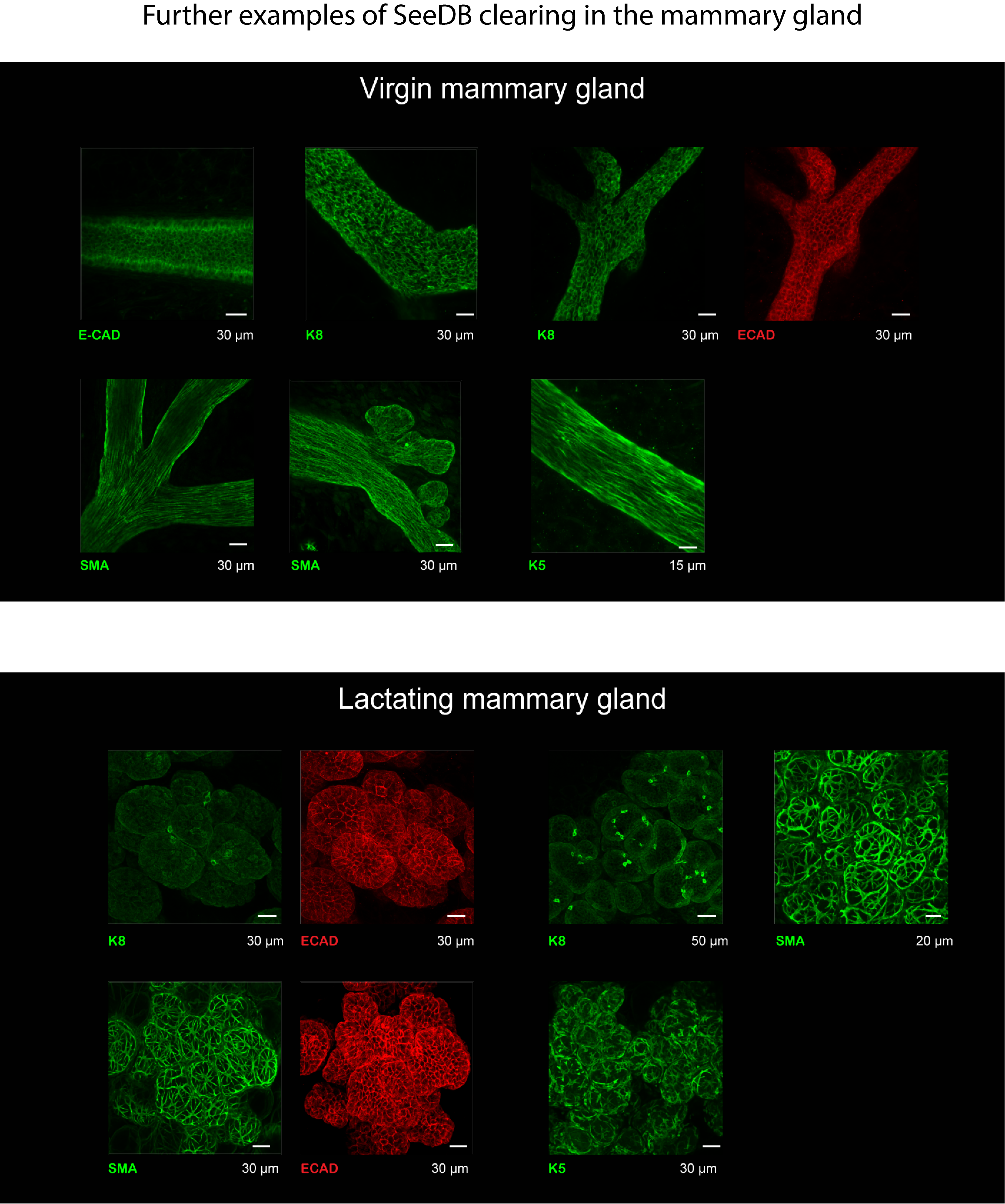

Supplement: Additional file 18: — All figures in high resolution. (ZIP 127 MB) [file 13058_2016_754_MOESM18_ESM.zip › Final final PNG for online links/Additional File 13 Fig S11.png]

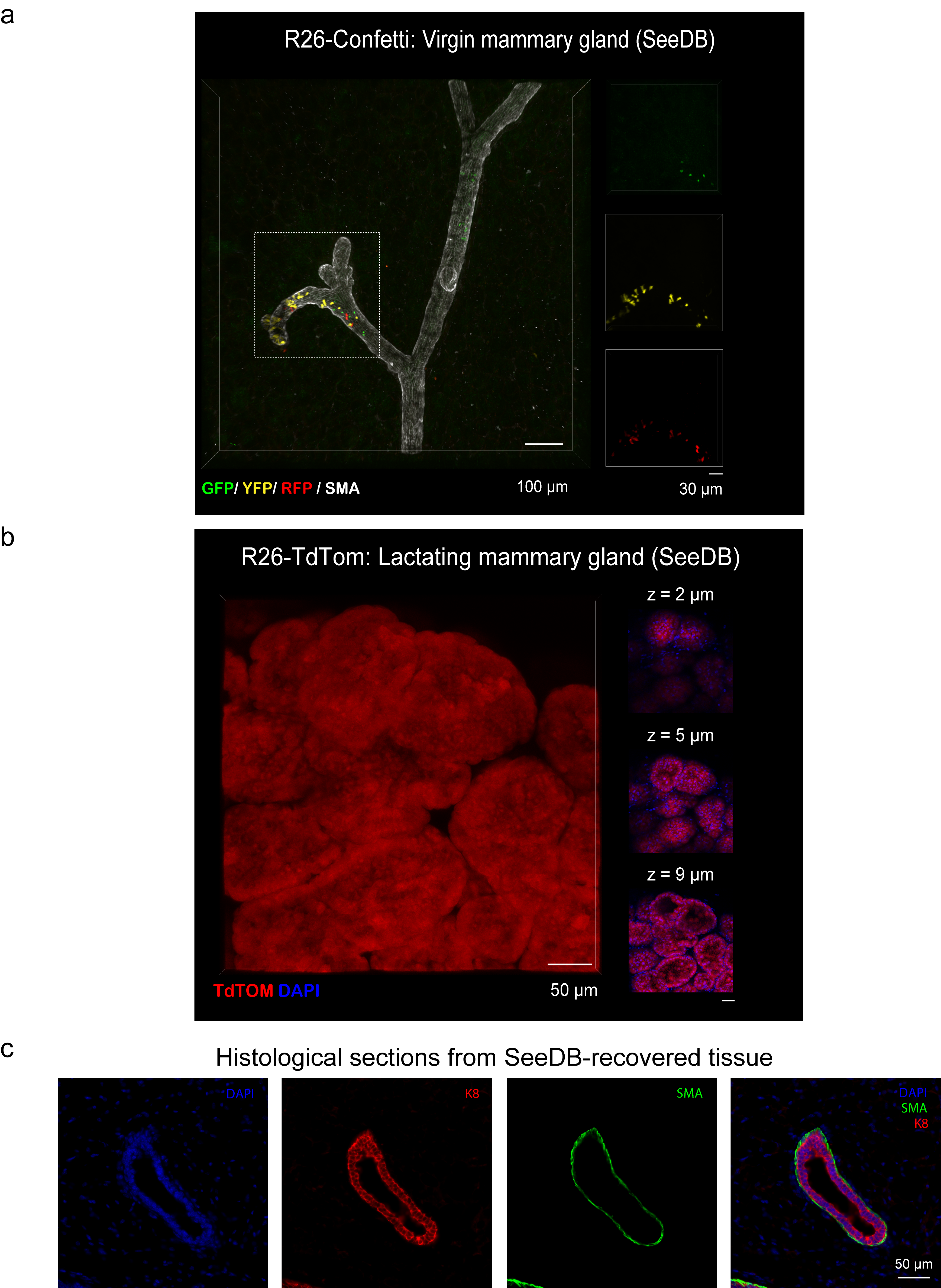

Supplement: Additional file 18: — All figures in high resolution. (ZIP 127 MB) [file 13058_2016_754_MOESM18_ESM.zip › Final final PNG for online links/Additional File 14 Fig S12.png]

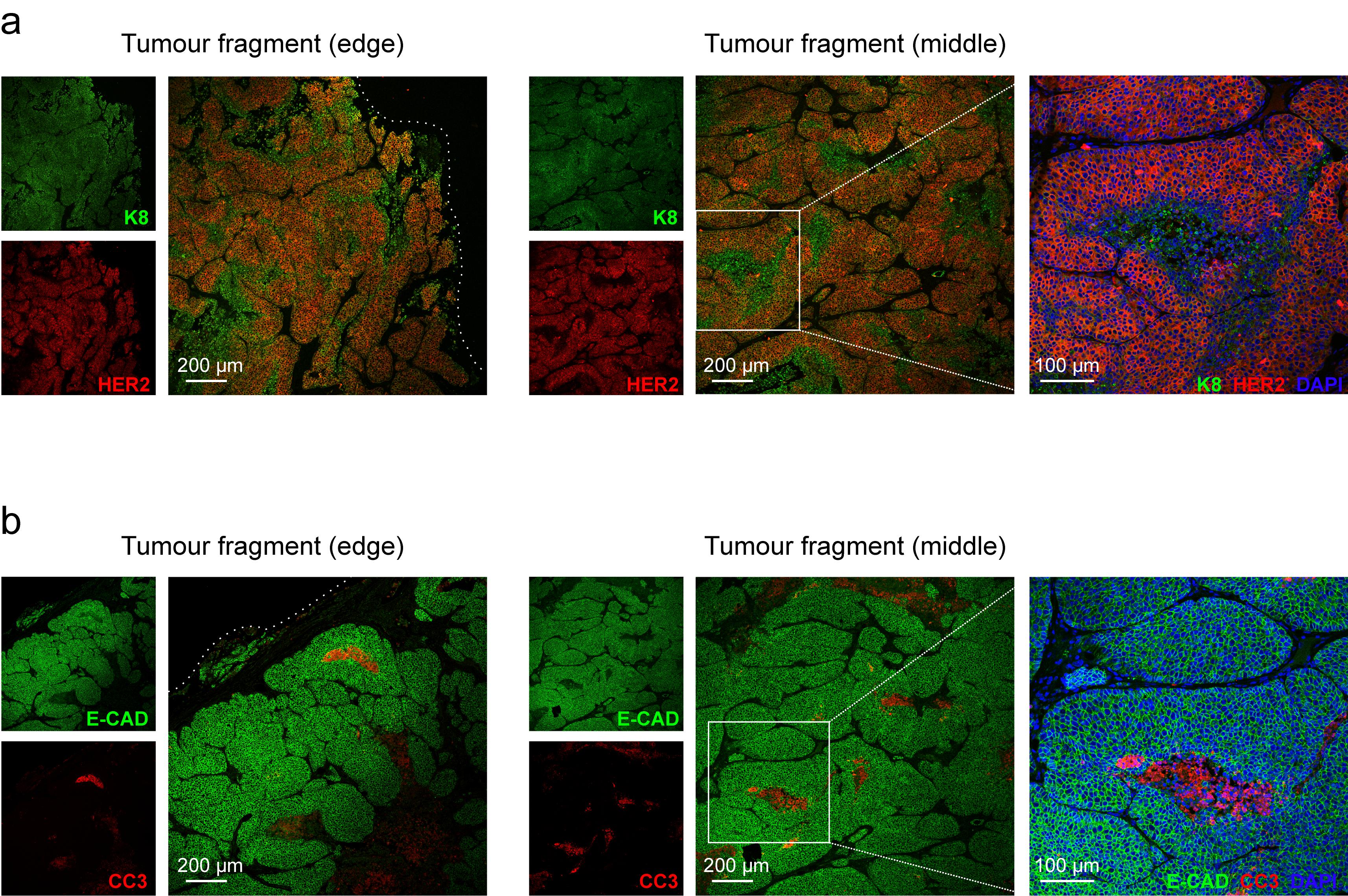

Supplement: Additional file 18: — All figures in high resolution. (ZIP 127 MB) [file 13058_2016_754_MOESM18_ESM.zip › Final final PNG for online links/Additional File 16 Fig S13.png]

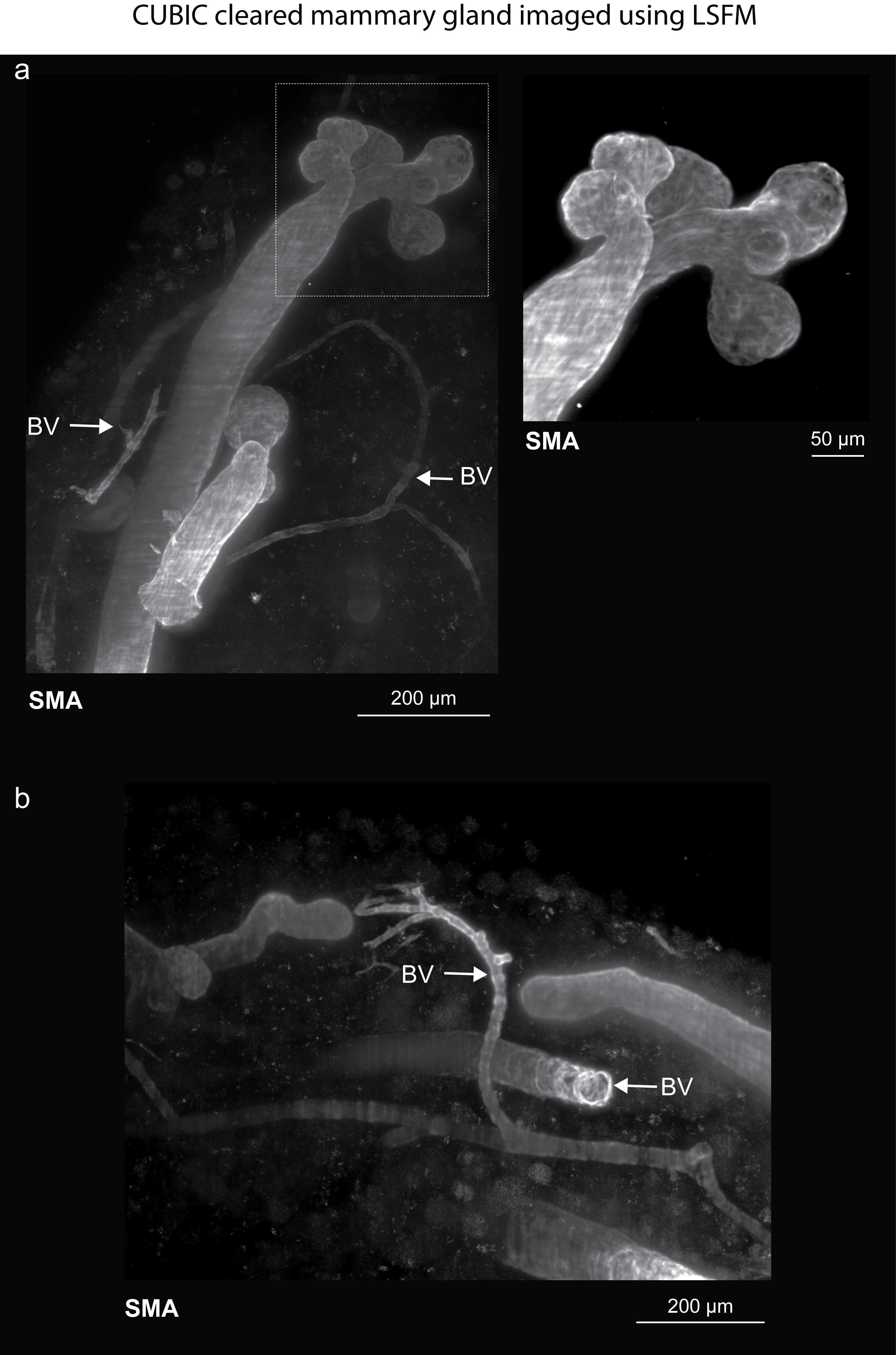

Supplement: Additional file 18: — All figures in high resolution. (ZIP 127 MB) [file 13058_2016_754_MOESM18_ESM.zip › Final final PNG for online links/Additional File 17 Fig S14.png]

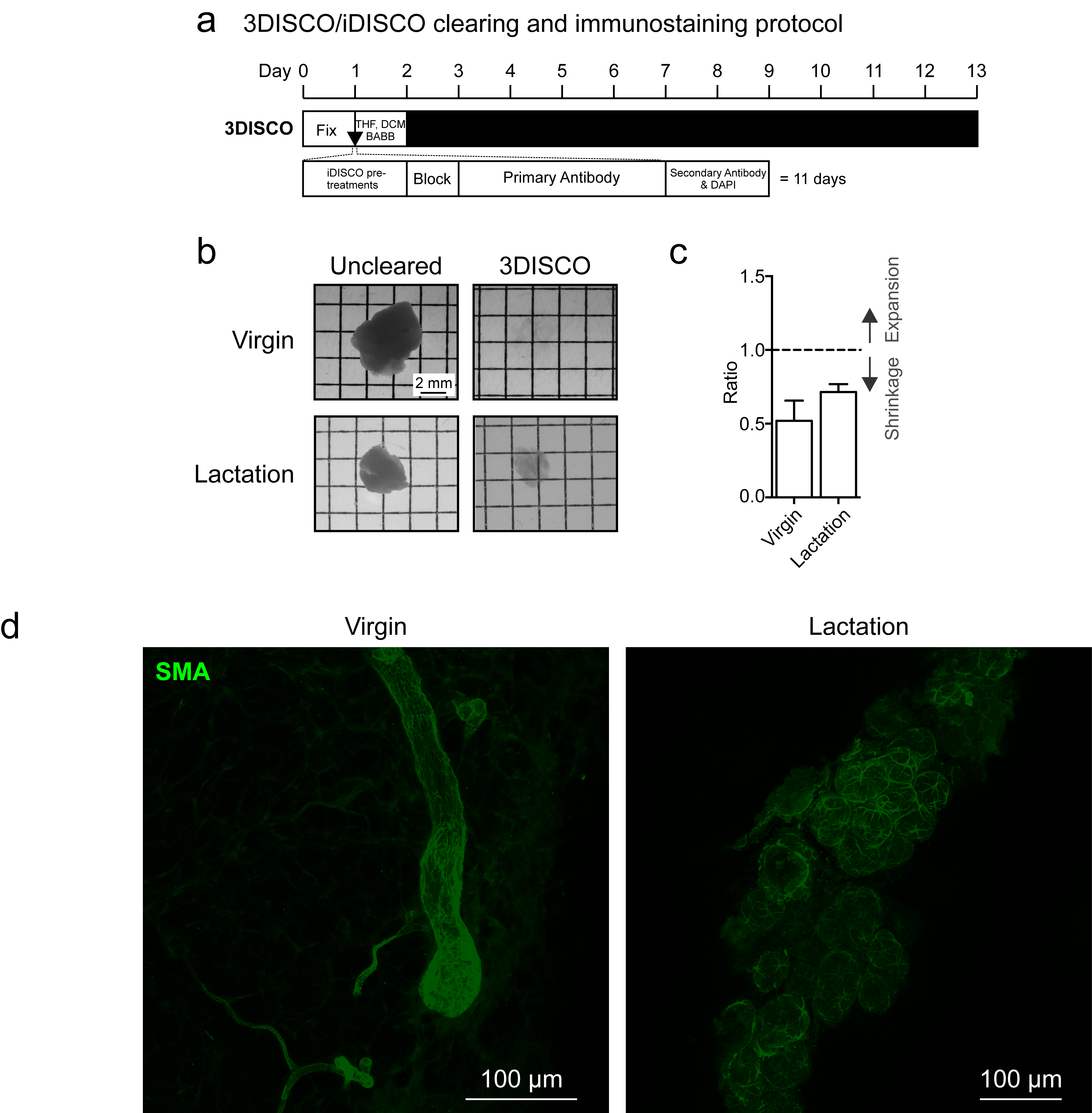

Supplement: Additional file 18: — All figures in high resolution. (ZIP 127 MB) [file 13058_2016_754_MOESM18_ESM.zip › Final final PNG for online links/Additional File 2 Fig S2.png]

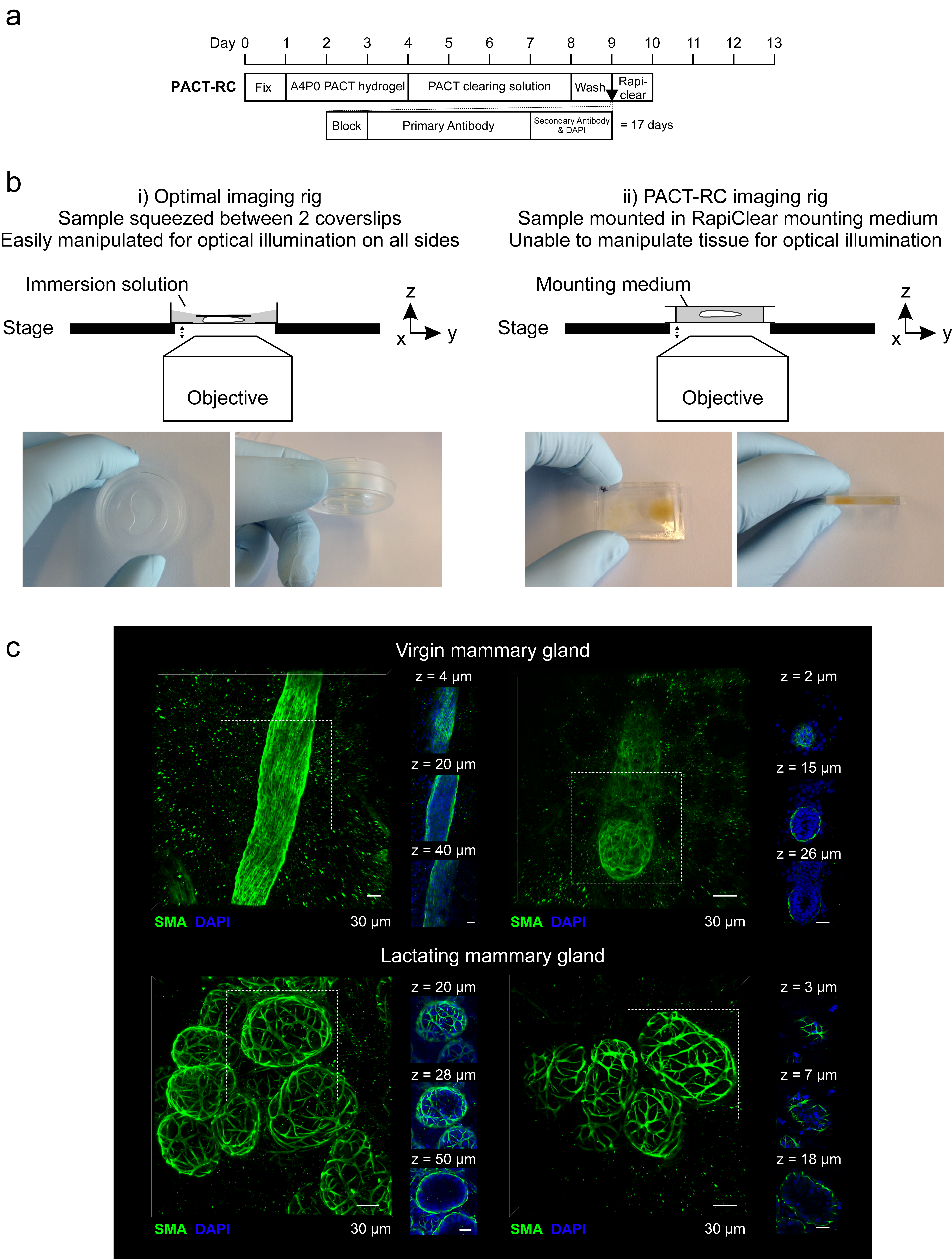

Supplement: Additional file 18: — All figures in high resolution. (ZIP 127 MB) [file 13058_2016_754_MOESM18_ESM.zip › Final final PNG for online links/Additional File 3 Fig S3.png]

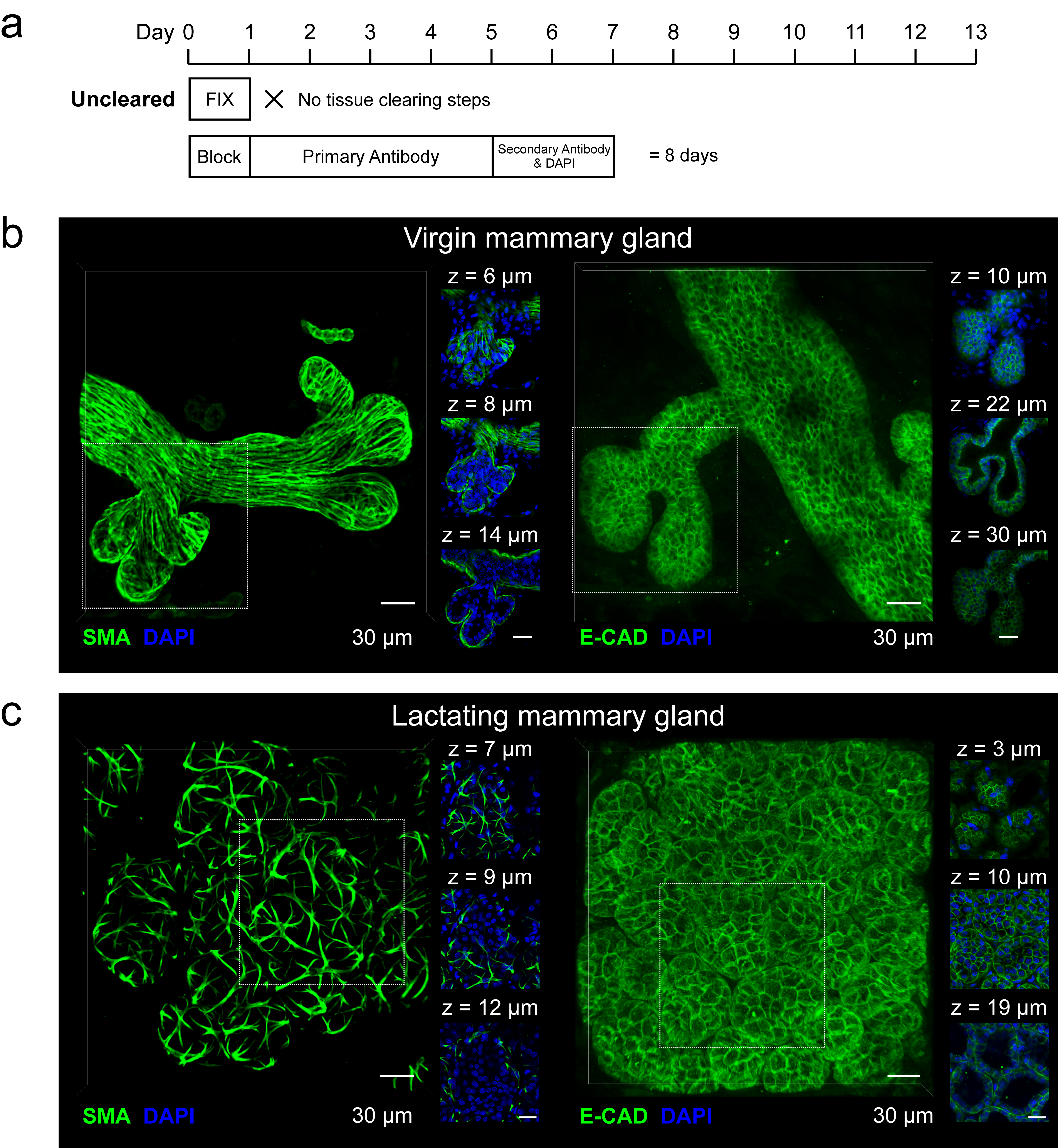

Supplement: Additional file 18: — All figures in high resolution. (ZIP 127 MB) [file 13058_2016_754_MOESM18_ESM.zip › Final final PNG for online links/Additional File 4 Fig S4.png]

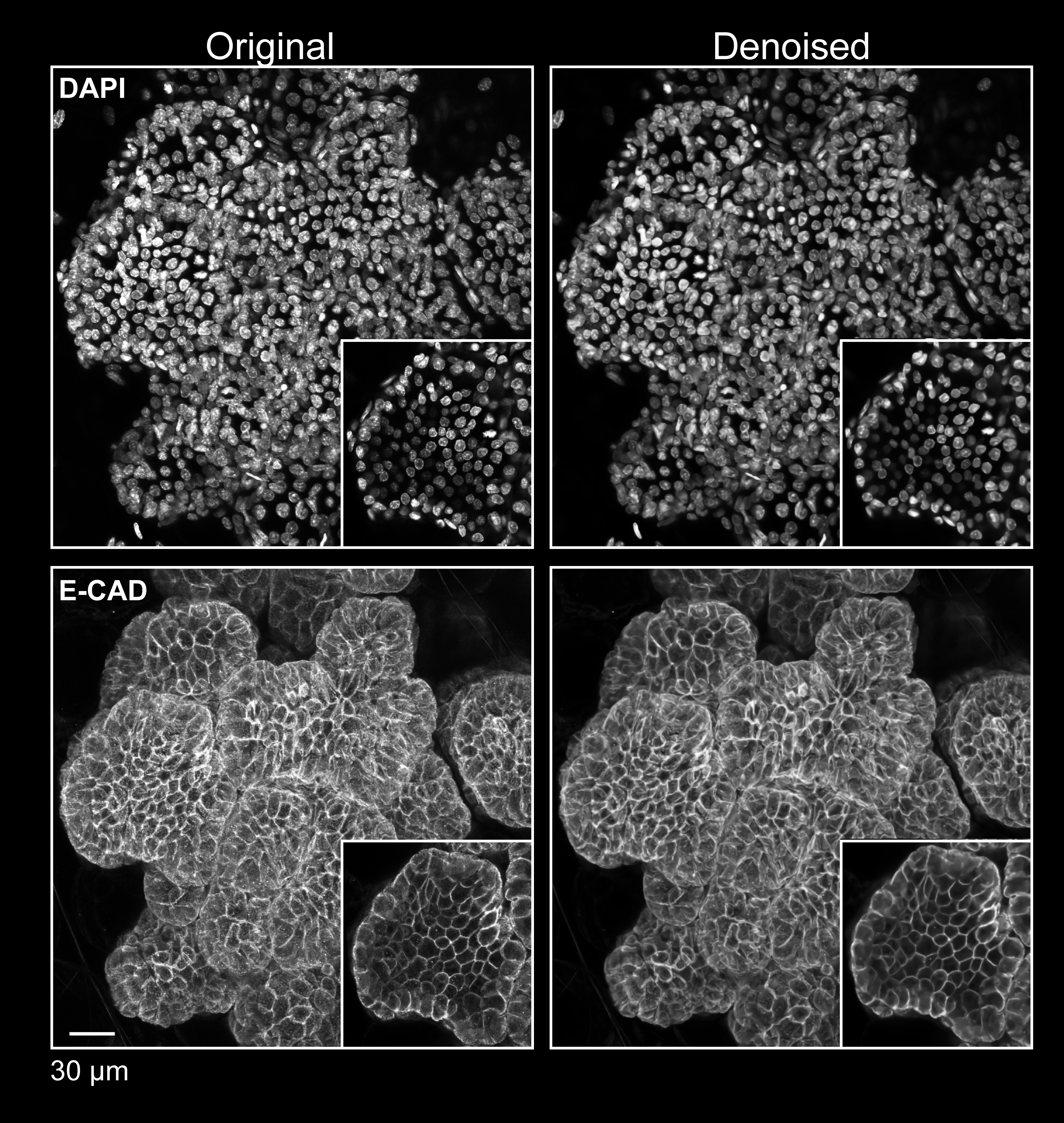

Supplement: Additional file 18: — All figures in high resolution. (ZIP 127 MB) [file 13058_2016_754_MOESM18_ESM.zip › Final final PNG for online links/Additional File 5 Fig S5.png]

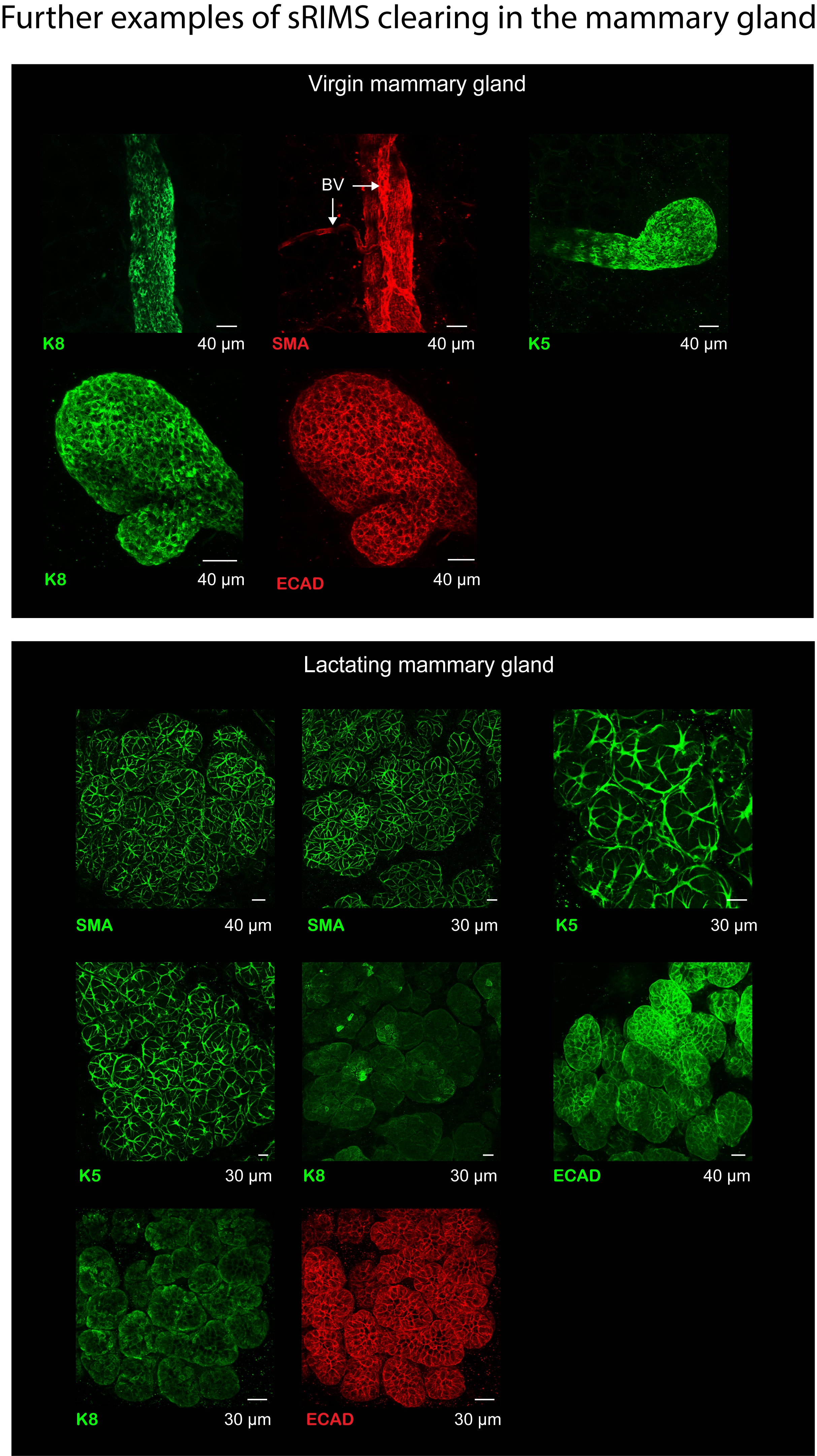

Supplement: Additional file 18: — All figures in high resolution. (ZIP 127 MB) [file 13058_2016_754_MOESM18_ESM.zip › Final final PNG for online links/Additional File 6 Fig S6.png]

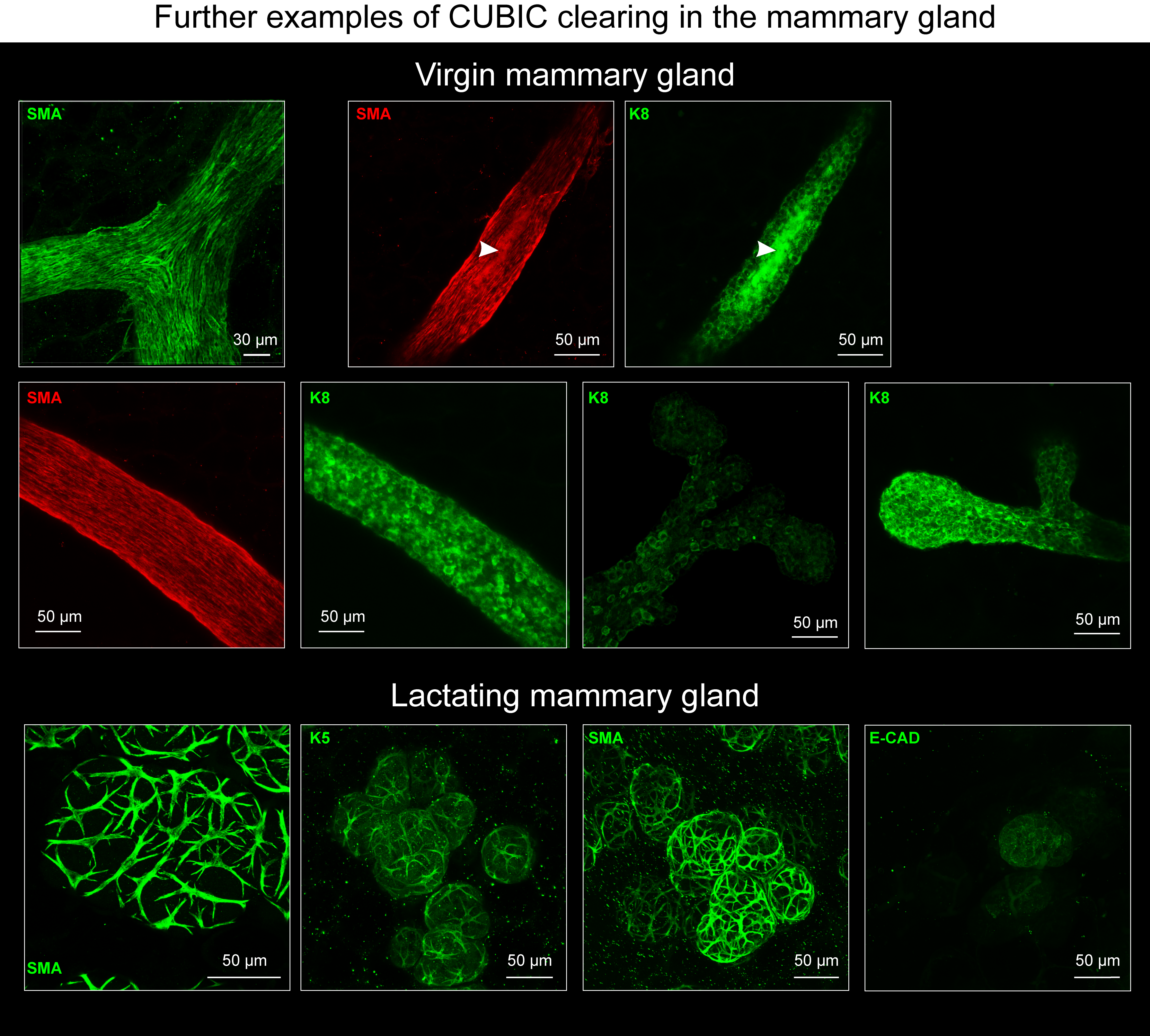

Supplement: Additional file 18: — All figures in high resolution. (ZIP 127 MB) [file 13058_2016_754_MOESM18_ESM.zip › Final final PNG for online links/Additional File 8 Fig S7.png]

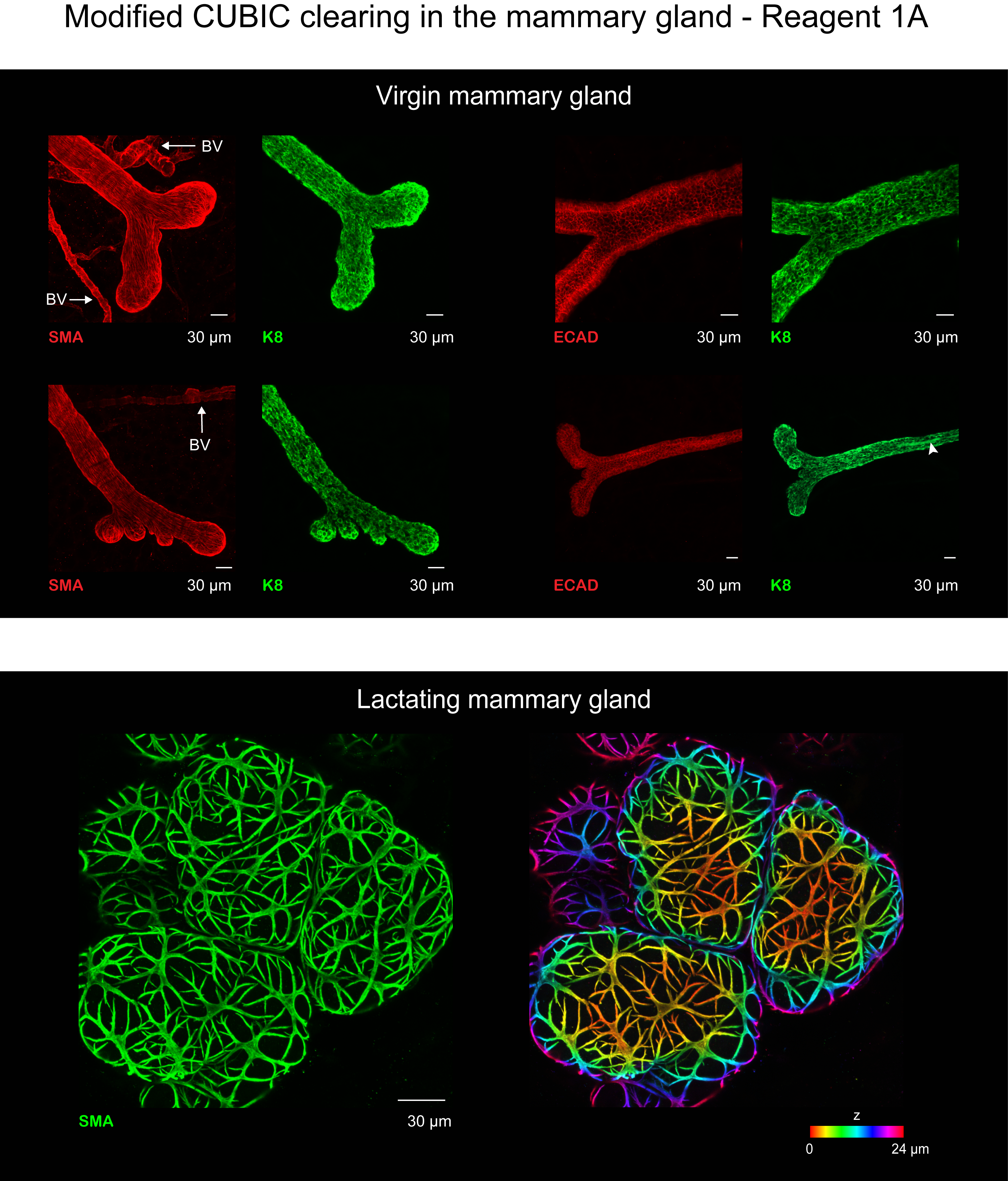

Supplement: Additional file 18: — All figures in high resolution. (ZIP 127 MB) [file 13058_2016_754_MOESM18_ESM.zip › Final final PNG for online links/Additional File 9 Fig S8.png]

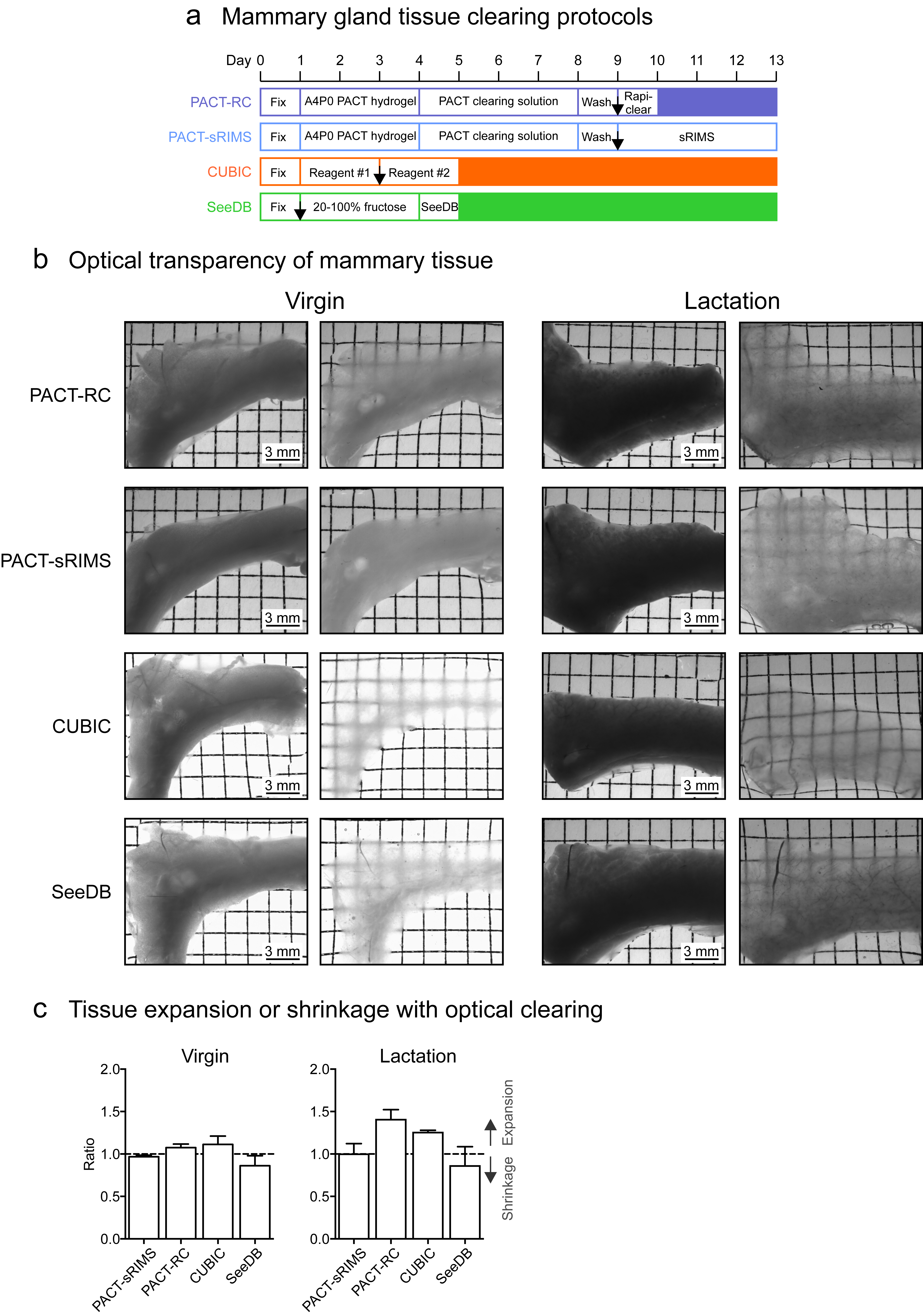

Supplement: Additional file 18: — All figures in high resolution. (ZIP 127 MB) [file 13058_2016_754_MOESM18_ESM.zip › Final final PNG for online links/Figure 1.png]

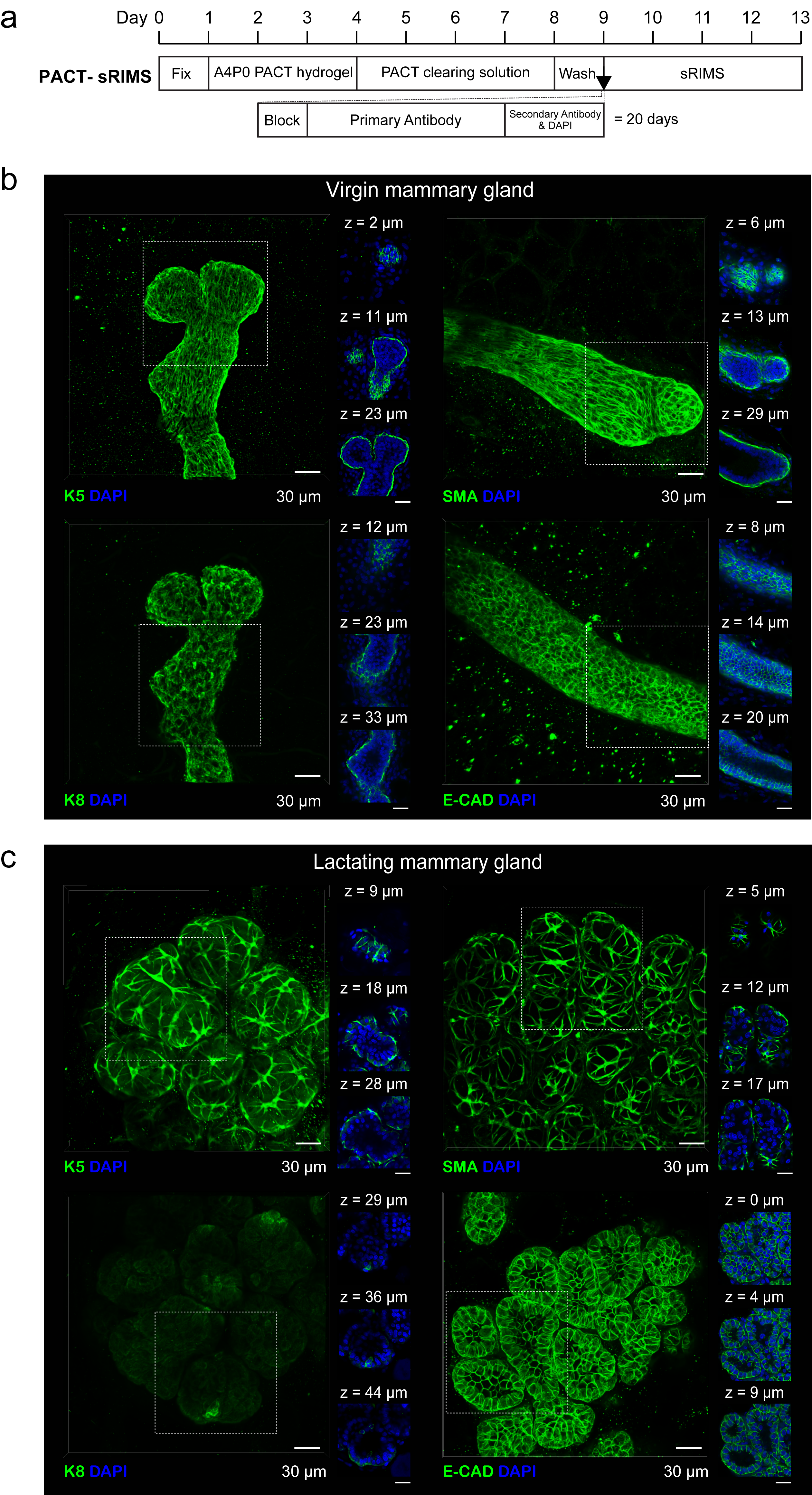

Supplement: Additional file 18: — All figures in high resolution. (ZIP 127 MB) [file 13058_2016_754_MOESM18_ESM.zip › Final final PNG for online links/Figure 2.png]

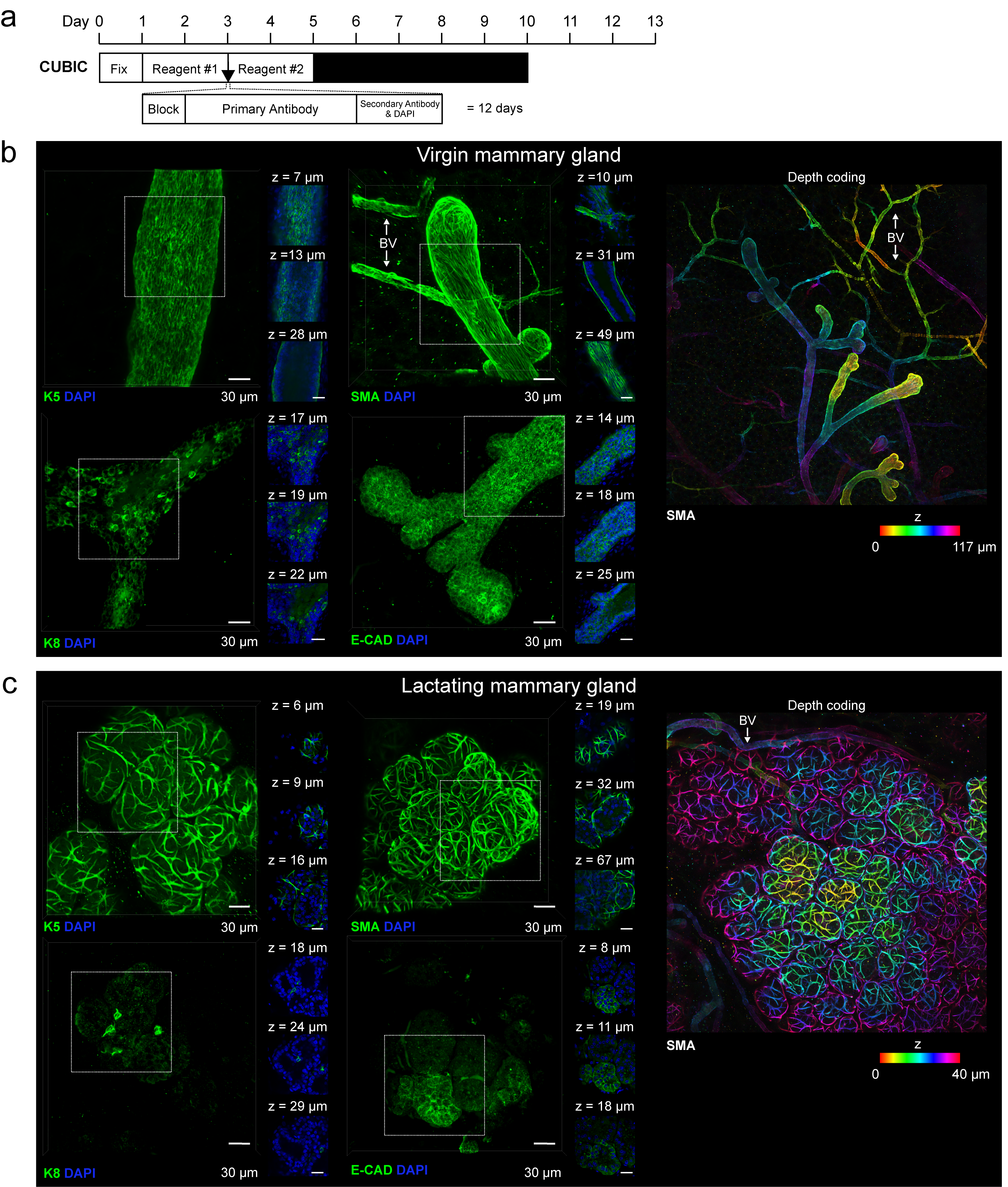

Supplement: Additional file 18: — All figures in high resolution. (ZIP 127 MB) [file 13058_2016_754_MOESM18_ESM.zip › Final final PNG for online links/Figure 3.png]

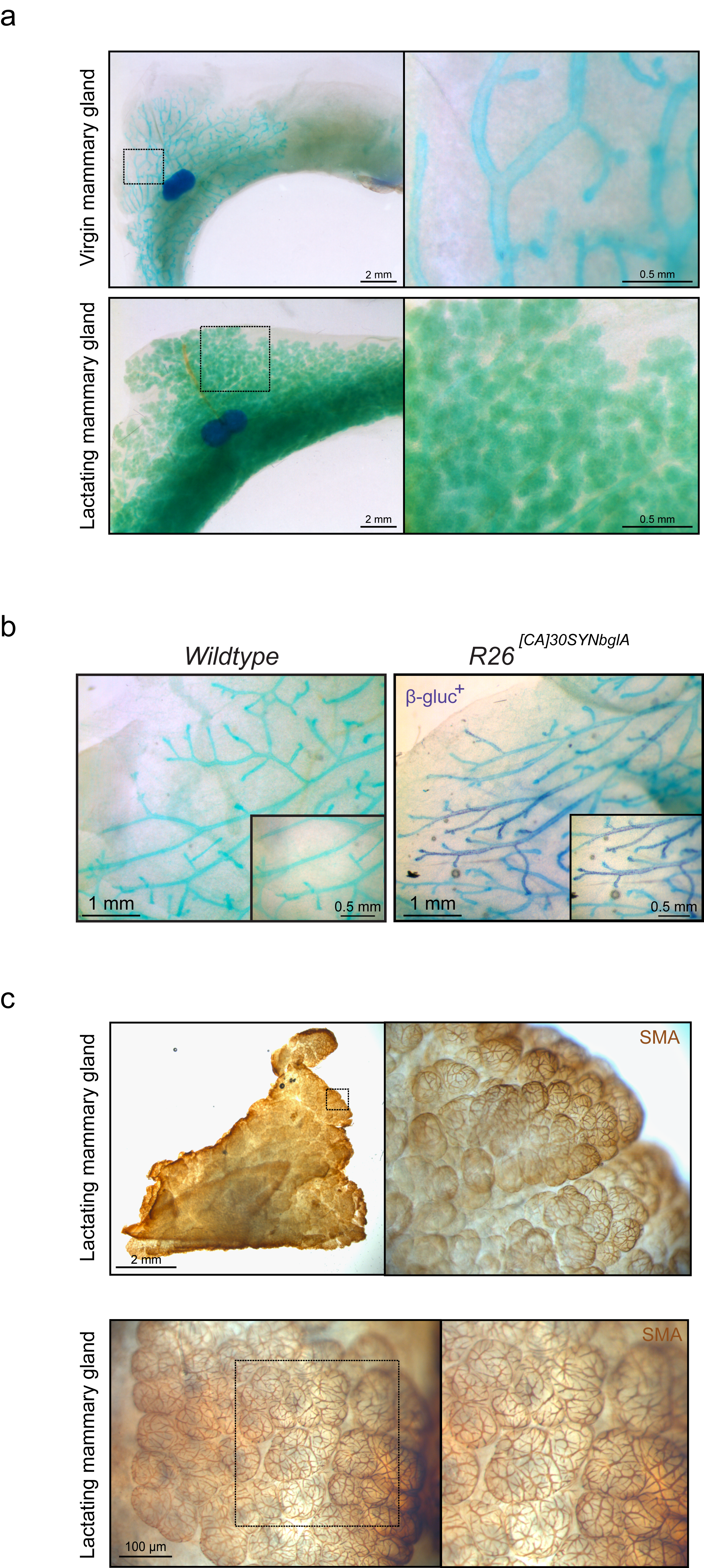

Supplement: Additional file 18: — All figures in high resolution. (ZIP 127 MB) [file 13058_2016_754_MOESM18_ESM.zip › Final final PNG for online links/Figure 4.png]

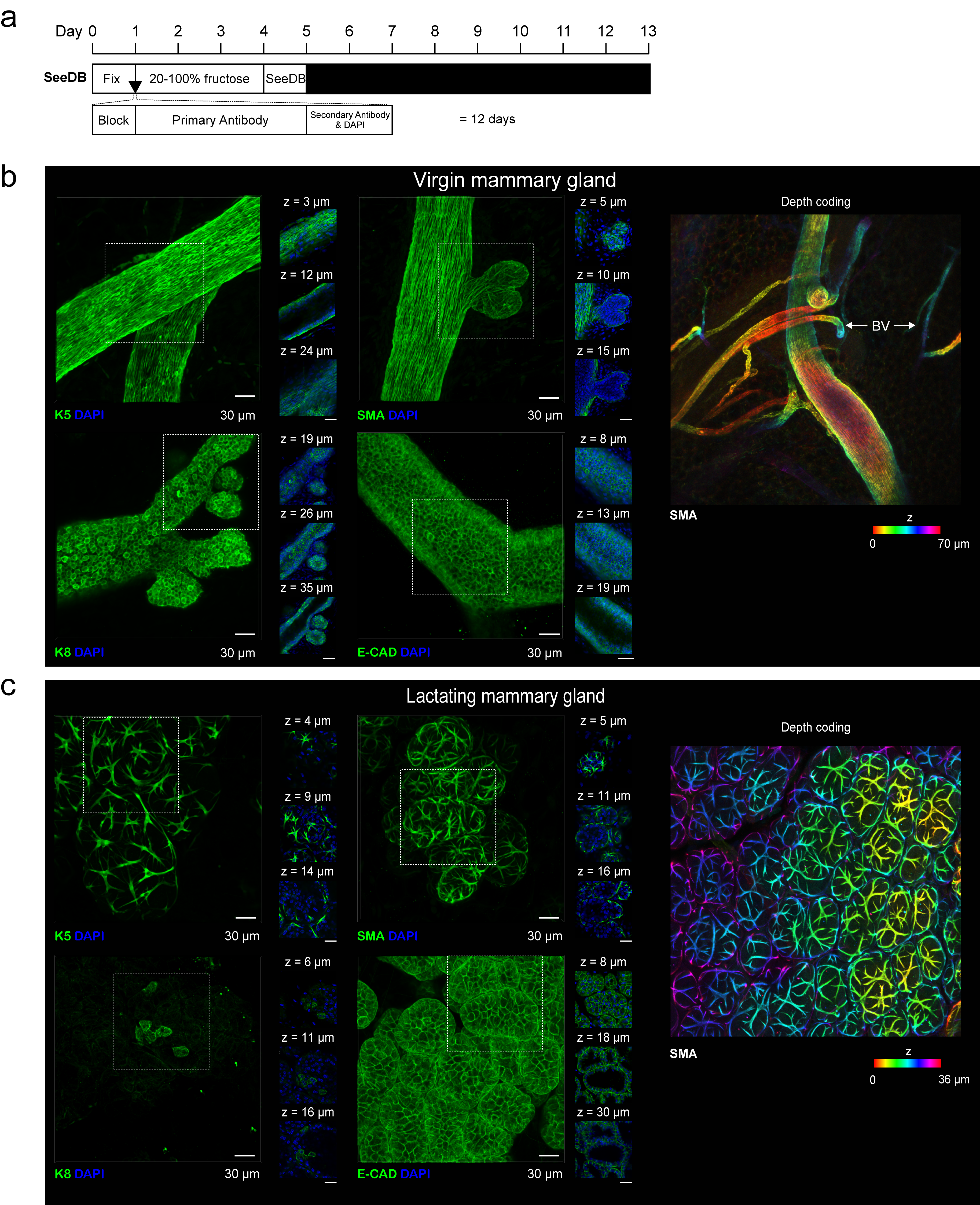

Supplement: Additional file 18: — All figures in high resolution. (ZIP 127 MB) [file 13058_2016_754_MOESM18_ESM.zip › Final final PNG for online links/Figure 5.png]

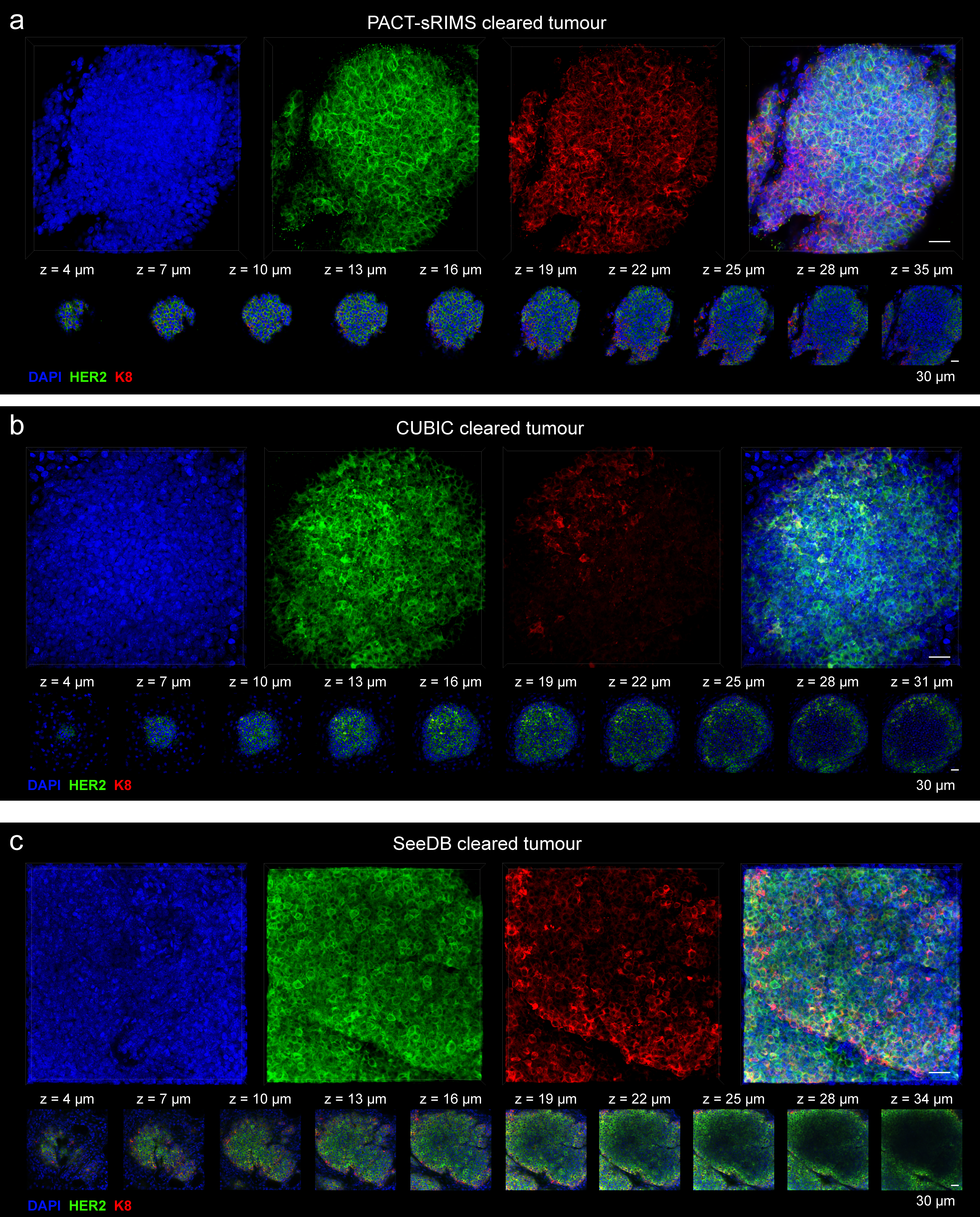

Supplement: Additional file 18: — All figures in high resolution. (ZIP 127 MB) [file 13058_2016_754_MOESM18_ESM.zip › Final final PNG for online links/Figure 6.png]

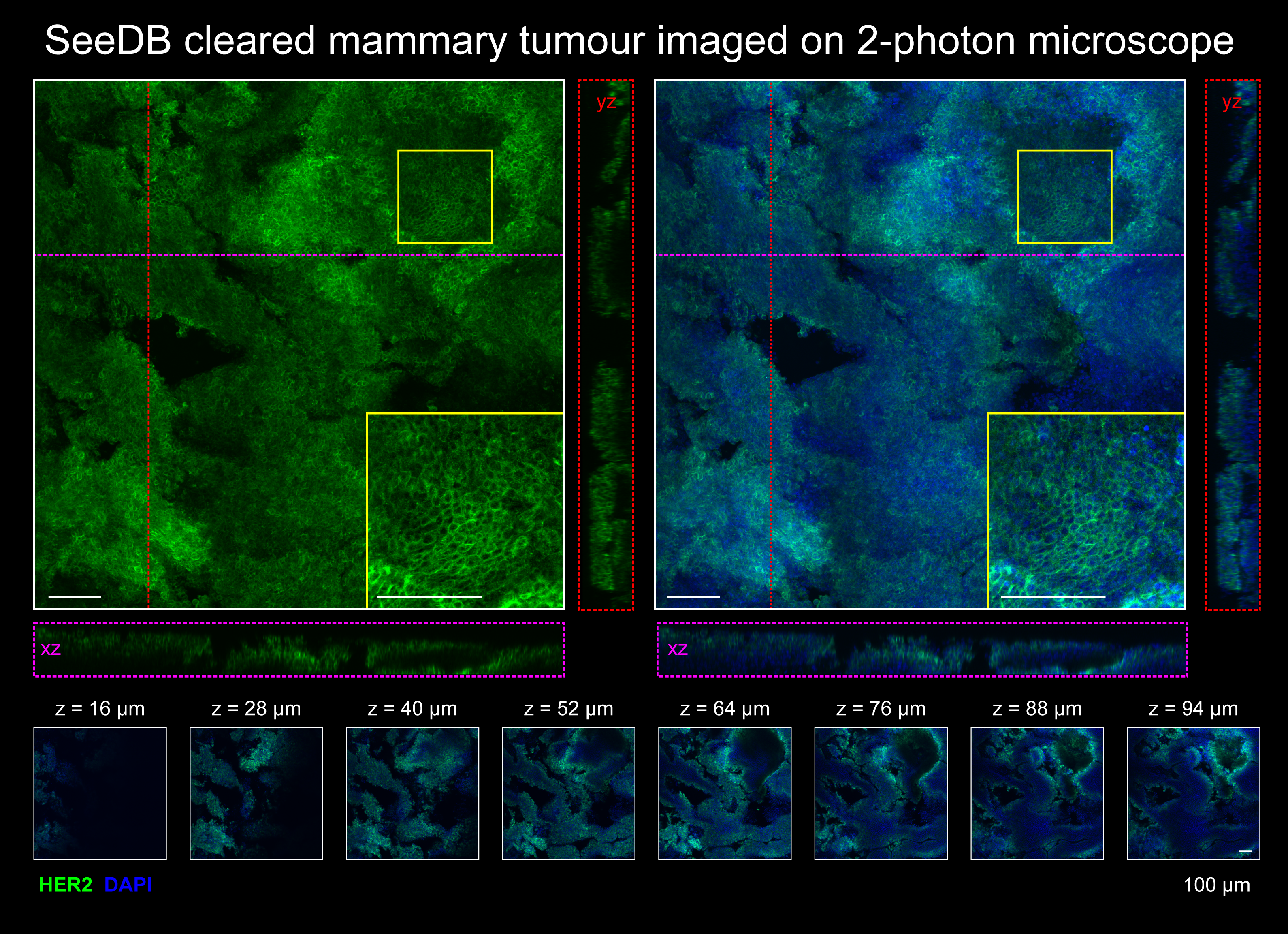

Supplement: Additional file 18: — All figures in high resolution. (ZIP 127 MB) [file 13058_2016_754_MOESM18_ESM.zip › Final final PNG for online links/Figure 7.png]
