# Supplementary material for: Imaging the mammary gland and mammary tumours in 3D: optical tissue clearing and immunofluorescence methods
Source: Breast Cancer Res. 2016 Dec 13;18:127. doi: 10.1186/s13058-016-0754-9 (PMC5155399; doi:10.1186/s13058-016-0754-9)
Supplement: Additional file 3: Figure S3. — PACT-RC clearing and 3D imaging of virgin and lactating mouse mammary tissue. a PACT-RC tissue clearing and immunostaining protocol and timeline. b The optimal (i) and PACT-RC (ii) imaging configuration for confocal microscopy. For optimal illumination, samples are squeezed between two glass coverslips and are easily flipped or re-positioned for imaging. In contrast, for PACT-RC-cleared samples, samples are mounted using an iSpacer chamber and are difficult to adjust or reposition for optimal illumination, thus the working distance becomes the limiting factor in image acquisition. To overcome this, sample thickness must be closely matched to the thickness of the iSpacer chamber, specialised imaging objectives need to be used or different RI-matching solutions are needed. c 3D confocal imaging of PACT-RC-cleared virgin and lactating mammary glands immunostained with the basal cell marker SMA and stained with the nuclear stain DAPI. These images are representative of images from at least two mice. See Additional file 18 for a high resolution version of these PDFs. (PDF 124 mb) [file 13058_2016_754_MOESM3_ESM.pdf]

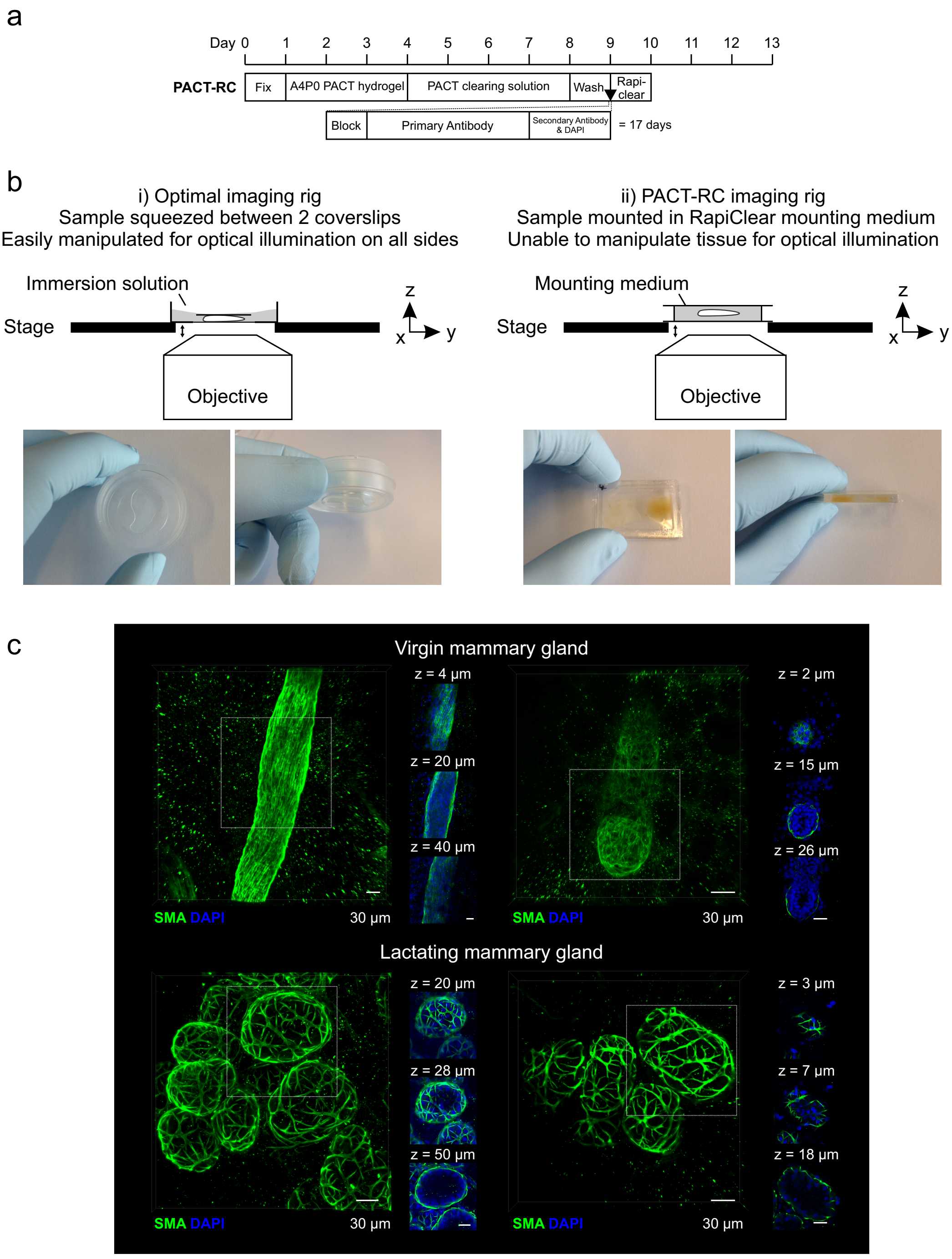
a
Day 0 1 2 3 4 5 6 7 8 9 10 11 12 13
PACT-RC
Fix
A4P0 PACT hydrogel
PACT clearing solution
Wash
Rapi-clear
Block
Primary Antibody
Secondary Antibody & DAPI
= 17 days
b
i) Optimal imaging rig
Sample squeezed between 2 coverslips
Easily manipulated for optical illumination on all sides
Immersion solution
Stage
Objective
z
x
y
ii) PACT-RC imaging rig
Sample mounted in RapiClear mounting medium
Unable to manipulate tissue for optical illumination
Mounting medium
Stage
Objective
z
x
y
c
Virgin mammary gland
z = 4 µm
z = 20 µm
z = 40 µm
SMA DAPI
30 µm
z = 2 µm
z = 15 µm
z = 26 µm
SMA DAPI
30 µm
Lactating mammary gland
z = 20 µm
z = 28 µm
z = 50 µm
SMA DAPI
30 µm
z = 3 µm
z = 7 µm
z = 18 µm
SMA DAPI
30 µm
