# Supplementary material for: Imaging the mammary gland and mammary tumours in 3D: optical tissue clearing and immunofluorescence methods
Source: Breast Cancer Res. 2016 Dec 13;18:127. doi: 10.1186/s13058-016-0754-9 (PMC5155399; doi:10.1186/s13058-016-0754-9)

Further examples of CUBIC clearing in the mammary gland

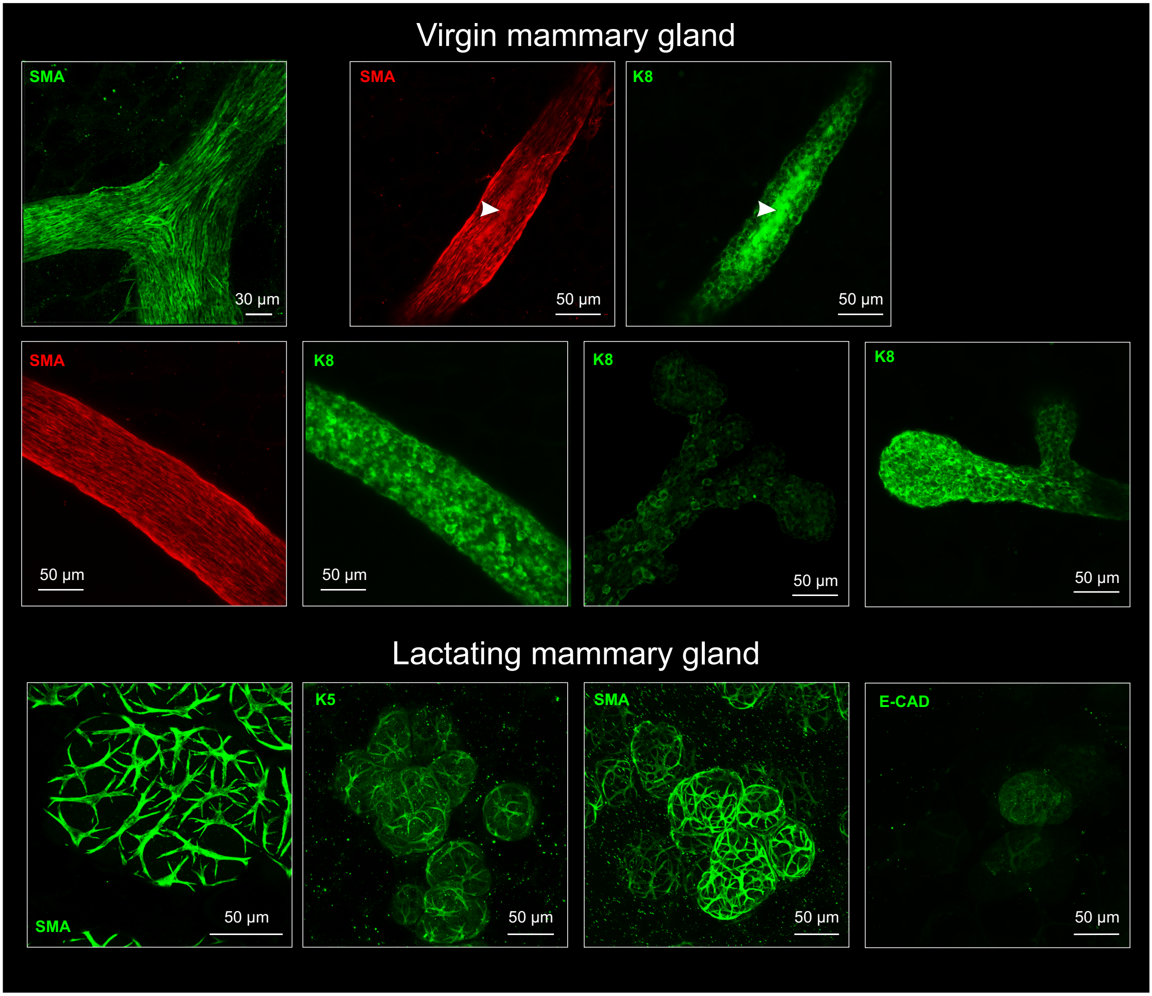

Supplement: Additional file 8: Figure S7. — Additional 3D confocal images of CUBIC-cleared mammary glands, related to Fig. 3. Arrowhead shows non-specific intraluminal staining occasionally observed with CUBIC clearing, which may be improved with more rigorous washing following immersion in Reagent 1. See Additional file 18 for a high resolution version of these PDFs. (PDF 23 mb) [file 13058_2016_754_MOESM8_ESM.pdf]
