# Supplementary material for: Imaging the mammary gland and mammary tumours in 3D: optical tissue clearing and immunofluorescence methods
Source: Breast Cancer Res. 2016 Dec 13;18:127. doi: 10.1186/s13058-016-0754-9 (PMC5155399; doi:10.1186/s13058-016-0754-9)
Supplement: Additional file 11: Figure S10. — Compatibility of CUBIC clearing with carmine and haematoxylin whole-mount staining. Carmine staining (top) shows light pink, non-uniform staining in virgin and lactating tissue. Haematoxylin staining (bottom) was an intense blue colour in both ducts and stroma. See Additional file 18 for a high resolution version of these PDFs. (PDF 30 mb) [file 13058_2016_754_MOESM11_ESM.pdf]

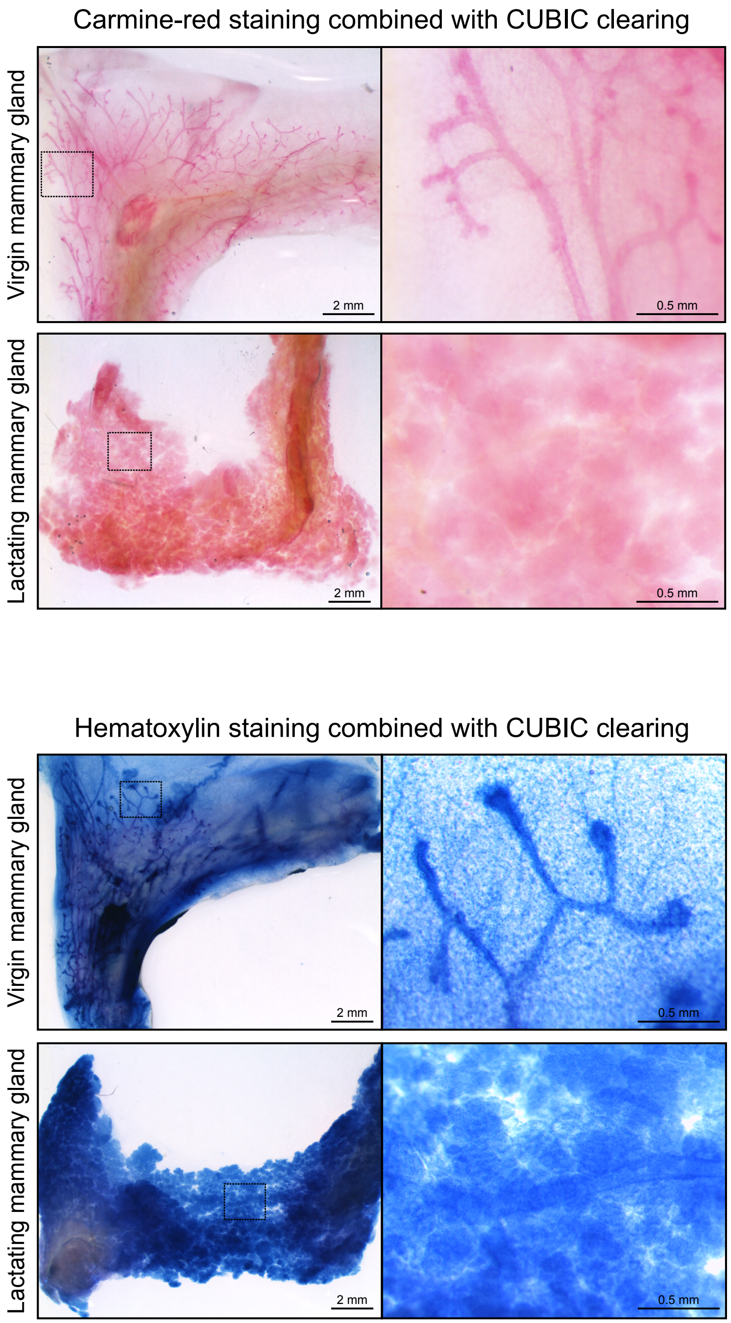
Carmine-red staining combined with CUBIC clearing
Virgin mammary gland
Lactating mammary gland
2 mm
0.5 mm
Hematoxylin staining combined with CUBIC clearing
Virgin mammary gland
Lactating mammary gland
2 mm
0.5 mm
