# Supplementary material for: Imaging the mammary gland and mammary tumours in 3D: optical tissue clearing and immunofluorescence methods
Source: Breast Cancer Res. 2016 Dec 13;18:127. doi: 10.1186/s13058-016-0754-9 (PMC5155399; doi:10.1186/s13058-016-0754-9)
Supplement: Additional file 1: Figure S1. — No primary antibody controls of CUBIC cleared tissue (top panel) and SeeDB cleared tissue (bottom panel). See Additional file 18 for a high resolution version of these PDFs. (PDF 243 mb) [file 13058_2016_754_MOESM1_ESM.pdf]

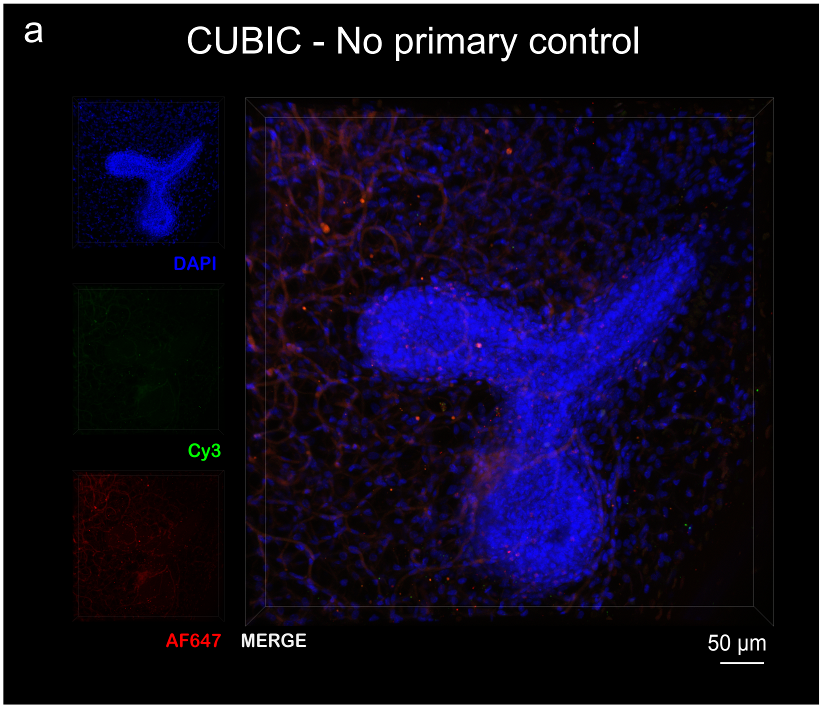
a
CUBIC - No primary control
DAPI
Cy3
AF647
MERGE
50 µm

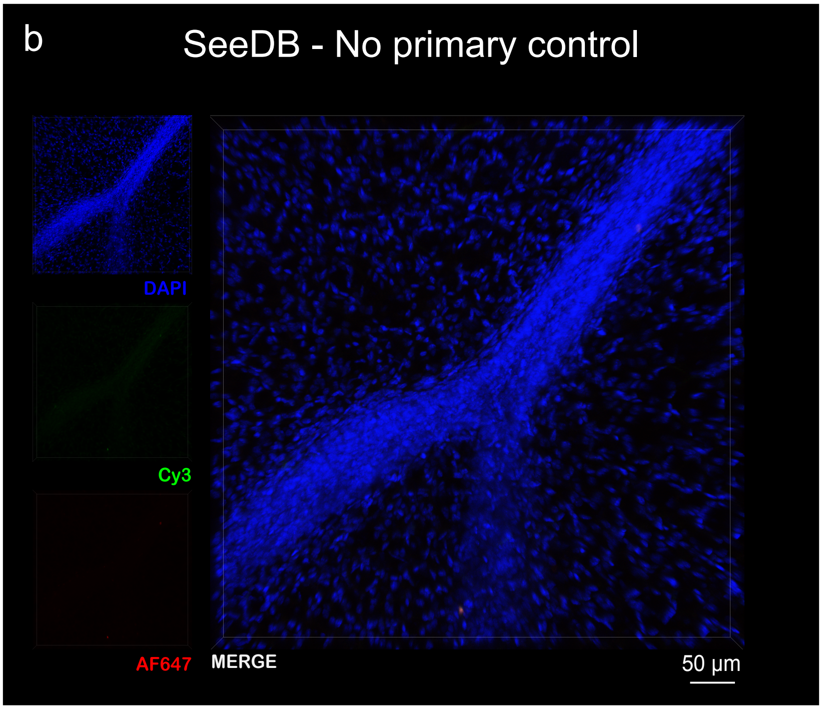
b
SeeDB - No primary control
DAPI
Cy3
AF647
MERGE
50 µm
